# My Bridge (*Mi Puente*), a care transitions intervention for Hispanics/Latinos with multimorbidity and behavioral health concerns: protocol for a randomized controlled trial

**DOI:** 10.1186/s13063-019-3722-8

**Published:** 2020-02-12

**Authors:** Linda C. Gallo, Addie L. Fortmann, Julia I. Bravin, Taylor L. Clark, Kimberly L. Savin, Duvia Lara Ledesma, Johanna Euyoque, Haley Sandoval, Scott C. Roesch, Todd Gilmer, Gregory A. Talavera, Athena Philis-Tsimikas

**Affiliations:** 1 Department of Psychology, San Diego State University, San Diego CA, USA; 2 Scripps Whittier Diabetes Institute, Scripps Health, La Jolla CA, USA; 3 Joint Doctoral Program in Clinical Psychology, San Diego State University/University of California, San Diego, San Diego, CA USA; 4 San Diego State University Research Foundation, San Diego CA, USA; 5 Department of Family Medicine and Public Health, University of California, San Diego, USA

**Keywords:** Clinical trial, Health behavior, Hispanic Americans, Mental health, Multimorbidity, Patient readmission, Transitional care

## Abstract

**Background:**

Multimorbidity affects four of ten US adults and eight of ten adults ages 65 years and older, and frequently includes both cardiometabolic conditions and behavioral health concerns. Hispanics/Latinos (hereafter, Latinos) and other ethnic minorities are more vulnerable to these conditions, and face structural, social, and cultural barriers to obtaining quality physical and behavioral healthcare. We report the protocol for a randomized controlled trial that will compare *Mi Puente* (My Bridge), a cost-efficient care transitions intervention conducted by a specially trained Behavioral Health Nurse and Volunteer Community Mentor team, to usual care or best-practice discharge approaches, in reducing hospital utilization and improving patient reported outcomes in Latino adults with multiple cardiometabolic conditions and behavioral health concerns. The study will examine the degree to which *Mi Puente* produces superior reductions in hospital utilization at 30 and 180 days (primary aim) and better patient-reported outcomes (quality of life/physical health; barriers to healthcare; engagement with outpatient care; patient activation; resources for chronic disease management), and will examine the cost effectiveness of the *Mi Puente* intervention relative to usual care.

**Methods:**

Participants are enrolled as inpatients at a South San Diego safety net hospital, using information from electronic medical records and in-person screenings. After providing written informed consent and completing self-report assessments, participants randomized to usual care receive best-practice discharge processes, which include educational materials, assistance with outpatient appointments, referrals to community-based providers, and other assistance (e.g., with billing, insurance) as required. Those randomized to *Mi Puente* receive usual-care materials and processes, along with inpatient visits and up to 4 weeks of follow-up phone calls from the intervention team to address their integrated physical-behavioral health needs and support the transition to outpatient care.

**Discussion:**

The *Mi Puente* Behavioral Health Nurse and Volunteer Community Mentor team intervention is proposed as a cost-effective and culturally appropriate care transitions intervention for Latinos with multimorbidity and behavioral health concerns. If shown to be effective, close linkages with outpatient healthcare and community organizations will help maximize uptake, dissemination, and scaling of the *Mi Puente* intervention.

**Trial registration:**

ClinicalTrials.gov: NCT02723019. Registered on 30 March 2016.

## Background and rationale

Multimorbidity or the presence of more than one chronic condition affected four of ten US adults and eight out of ten adults ages 65 years and older in 2014 [1]. Multimorbidity is associated with premature death [2, 3], disability, poor functional status and quality of life [2, 4, 5], and high healthcare expenses [2, 6, 7]. Several studies in the elderly have demonstrated a near-exponential relationship between number of chronic conditions and healthcare costs [7], with hospital utilization expenditures contributing significantly to these trends [8, 9].

Cardiometabolic disorders such as hypertension, dyslipidemia, diabetes mellitus, heart diseases, and behavioral health concerns, including mood and anxiety disorders, are among the ten most prevalent chronic conditions in US adults [1]. Racial/ethnic minorities including Hispanics/Latinos (hereafter, Latinos) have a higher prevalence of many of these conditions, particularly certain subpopulations such as Latinos of Mexican and Puerto Rican heritage, those who are more acculturated or have lived in the USA the longest, and those with lower socioeconomic status (SES) [10–14]. Latinos with diabetes mellitus and related conditions have more frequent complications and hospitalizations, greater functional impairment, lower quality of life, and higher mortality rates when compared to non-Latino whites [15, 16].

Latinos are more likely to experience serious psychological distress [17], yet less likely to have their behavioral health needs addressed when compared to non-Latino whites [18–21]. Even in the general population, behavioral health concerns are seriously undertreated with only about four in ten adults with a mental illness receiving mental healthcare in 2016 [22]. In addition to a lack of insurance coverage, stigma, cultural factors, and language barriers can impede mental healthcare access among ethnic/racial minorities [23–25]. Unfortunately, when left untreated, mental health problems have grave implications for both quantity and quality of life. Life expectancy for individuals with a serious mental illness is 13–30 years lower than in those without such conditions [26], a pattern often linked to treatable conditions and risk factors like smoking and obesity [27]. Undertreatment of behavioral health conditions also drains the US healthcare system. Mental illnesses, including depression, represent the third most frequent cause of hospitalization in US adults ages 18–44 years [28]. Among the costliest conditions in 2012, more expenses were incurred by treatment of mental disorders (US$29.6 million) than for any other condition [29].

Individuals with multimorbidity have a two to threefold increased risk of depression [30]. In particular, mental health and cardiometabolic conditions frequently co-occur [31–34]. When present, this mental-physical disease multimorbidity predicts poorer outcomes and higher healthcare costs [31, 35], in part due to longer hospital stays and more frequent readmissions [36–38]. Importantly, hospital readmissions have been identified as a central target for improving care coordination and reducing healthcare costs in the context of healthcare reform. For Medicare alone, reducing preventable readmissions by even 10% would result in an estimated US$1 billion in healthcare savings [39].

Evidenced-based care transitions services, including patient education, medication reconciliation, follow-up phone calls, and assistance with scheduling outpatient care, can help reduce readmissions among at-risk patients. A 2016 systematic review of 30 structured discharge programs revealed positive effects on readmissions and length of stay, and on patient and provider satisfaction with care; however, cost savings (when evaluated) were not evident [40]. Another recent review found that comprehensive discharge planning reduced 30-day readmission rates in medical-surgical patients [41]. Importantly, research into the effects of such programs on patient-reported health outcomes (e.g., quality of life) is scarce, highlighting an important evidence gap. In addition, these interventions may not adequately address the specific needs of low-income, ethnic/racial-minority individuals and those with behavioral health concerns, who are at high risk of readmission. Because many programs rely on nursing, pharmacy, and other relatively highly paid staff, and often incorporate resource-intensive home visits, they may not be maximally scalable and cost effective. Although systematic reviews have reported positive effects of integrating behavioral healthcare into primary care settings [42, 43], research concerning integrated care approaches in medical *inpatient* settings is limited to a few smaller studies that provide only preliminary evidence of improved outcomes, such as reduced length of stay [44]. No prior program, to our knowledge, has been developed to focus specifically on chronic cardiometabolic conditions, which are highly prevalent and associated with substantial patient and healthcare system burden. Further, much of the research on care transitions has neglected the systemic and ecological nature of the transitions process, focusing more heavily on patient knowledge and skills. A multi-level or social-ecological approach that acknowledges the interaction between the individual and his/her environment is needed to better guide provision of care for the highest-risk populations.

“My Bridge (*Mi Puente*) to Better Cardiometabolic Health and Well-being” is a randomized controlled trial of a culturally appropriate, interdisciplinary care transitions approach designed to support at-risk Latino adults pre and post hospital discharge as they navigate the barriers that contribute to inequities in healthcare access and perpetuate disparities in cardiometabolic and behavioral health. *Mi Puente* builds on a sustainable behavioral health nurse plus volunteer community mentor team-care model and strong, collaborative relationships between inpatient, outpatient, and community services, to meet the integrated (i.e., physical and behavioral) health needs of Latinos hospitalized with cardiometabolic-behavioral condition multimorbidity. Our randomized controlled trial will test the effectiveness of *Mi Puente* versus usual care (UC) - i.e., best-practice discharge procedures - in reducing hospital utilization, and improving patient-reported and cost-effectiveness outcomes. The chosen comparator will elucidate the extent to which *Mi Puente* is superior to more general evidence-based approaches that are designed to enhance care coordination for individuals with complex needs who are at risk of readmission. Ultimately, we seek to evaluate a culturally appropriate, sustainable, and scalable program that effectively addresses integrated health needs and reduces disparities in Latinos, with potential for generalizability to other at-risk populations.

### Study aims

The primary objective of the current study is to determine the effectiveness of the *Mi Puente* care transitions intervention versus UC in reducing 30-day and 180-day hospital utilization in Latinos with multiple cardiometabolic conditions and one or more behavioral health concerns, who are hospitalized at a large safety-net hospital in South San Diego County.

The secondary aims are:

1. To test the effectiveness of *Mi Puente* versus UC in improving patient-reported quality of life/physical health across 180 days.
2. To test the effectiveness of *Mi Puente* versus UC in reducing barriers to health care, and increasing engagement with outpatient care, patient activation, and resources for chronic disease management, across 180 days.
3. To examine the cost-effectiveness of *Mi Puente* versus UC.
4. Guided by the reach, efficacy, adoption, implementation, and maintenance (RE-AIM) framework [45], examine the success of the *Mi Puente* program in:

- Reaching a representative population segment (reach);
- Achieving meaningful outcomes through a well-implemented intervention (efficacy/implementation); and
- Creating an intervention that can be adopted by and maintained in a real-world environment (adoption/maintenance).

### Trial design

This is a randomized, controlled, single-blind parallel-group, superiority trial. Due to the nature of the intervention, participants are not blinded to condition. However, outcome assessors (i.e., individuals conducting medical records abstraction and participant interviews) are blinded to participants’ group assignments. The protocol has been developed in accordance with Good Clinical Practice, Standard Protocol Items: Recommendation for Interventional Trials (SPIRIT) (See Additional file 2 SPIRIT 2013 checklist), and Consolidated Standards of Reporting Trials (CONSORT) 2013 guidelines.

## Methods: participants, interventions, and outcomes

### Primary study settings

The primary study setting and participant enrollment site is Scripps Mercy Hospital, Chula Vista, which serves the South region in San Diego County, CA, USA. With nearly 500,000 residents as of 2016, the South region of San Diego adjoins the USA/Mexico border, and is home to a large number of Latino and low-income residents [46]. Relative to the broader San Diego County population, which is 33% Latino, the South region is 60.5% Latino. The patient population of Scripps Mercy Hospital, Chula Vista is 65% Latino. The hospital has 156 beds and more than 700 employees, and includes a 24-h emergency department, and intensive care unit and laboratory.

### Community partners

#### Outpatient healthcare system

To ensure that we meet the needs of and address gaps experienced by typical receiving ambulatory healthcare settings, we have partnered with a large, federally qualified community health center (FQHC) that serves many low-income, uninsured, and minority residents of the South San Diego region. The FQHC partner has been actively engaged in planning the research and developing and implementing the *Mi Puente* intervention, and will be a key collaborator in efforts to scale and disseminate the research.

#### Chula Vista Community Collaborative

The mission of the Chula Vista Community Collaborative (CVCC) is “enhancing community partnerships to develop and implement coordinated strategies and systems for future generations.” The CVCC works to integrate existing resources and assets to develop coordinated strategies and systems that promote the health, safety, and wellness of local residents. For the current study, CVCC members have consulted on study components such as participant recruitment, intervention materials, and methods for disseminating study findings. In particular, the CVCC has provided significant input on the *Community Resource Manual*, used by volunteer community mentors.

#### Scripps Mercy Hospital Chula Vista Well-Being Center

The Scripps Mercy Hospital Chula Vista Well-Being Center (CVWBC) provides Scripps patients and the community with health and wellness resources, support groups and health promotion education. The mission of the CVWBC is to improve access to and quality of care, increase health awareness, and guide services for the underserved. The CVWBC offers a wide variety of programs in senior health, maternal and child health, and chronic disease education, support, and management. The CVWBC is also committed to supporting Scripps Mercy patients post-discharge to help reduce readmissions and aid in their continuum of care. For the current trial, the CVWBC refers members to consult on study and intervention components, has provided input on the community resource manual, and is listed in the community resource manual as a resource for patients in need of more intensive support.

#### Community Advisory Board

*Mi Puente* study and intervention significance, design, and implementation have been informed by ongoing input from a Community Advisory Board (CAB) composed of diverse stakeholders. The CAB includes representation by Scripps Health and outpatient healthcare system personnel, CVWBC and CVCC staff, and Scripps Mercy hospital patients. The CAB met yearly during the initial project years and has received updated study reports by email. In the final year of the trial, we will conduct in-person CAB meetings to discuss study findings, dissemination to stakeholders, and methods to sustain, disseminate, and scale the intervention.

### Eligibility criteria

The target population for this trial is Spanish-speaking and/or English-speaking Latino adult patients (ages 18 years and older), hospitalized at Scripps Mercy Hospital for any reason, with two or more cardiometabolic conditions (including obesity, diabetes mellitus, hypertension, dyslipidemia, ischemic heart disease, congestive heart failure, peripheral vascular disease, stroke, or other chronic coronary conditions; see Table 1 for eligible International Statistical Classification of Diseases and Related Health Problems (ICD) 10 codes). Participants are also required to have at least one behavioral health concern, defined broadly to include psychological distress (elevated depression or anxiety symptoms, or disease-related distress), chronic stress in central life domains, health risk behaviors (smoking, at-risk levels of alcohol consumption), medication non-adherence, and/or lack of outpatient healthcare access and regular preventive visits. Exclusion criteria include pregnancy; a serious life-threatening condition with life expectancy < 6 months; psychiatric morbidity or cognitive impairment that precludes informed consent or intervention participation; discharge to a location other than home (e.g., skilled nursing facility (SNF)); and language other than Spanish or English. In addition, participants without access to a telephone are excluded, since part of the intervention is delivered by telephone.

**Table 1:** International Statistical Classification of Diseases (ICD)-Version 10 codes utilized to identify patients eligible for the *Mi Puente* trial

Cardiometabolic condition	ICD 10 code ranges
Peripheral vascular disease (PVD)	443.9–443.99
	441.0–441.99
	785.4–785.49
	V43.4 - V43.49
	I71.0 - I71.999
	I79.0 - I79.099
	I73.1 - I73.199
	I73.8 - I73.899
	I73.9 - I73.999
	R02.0 - R02.999
	Z95.8 - Z95.899
	Z95.9 - Z95.999
	K55.1 - K55.199
	K55.8 - K55.899
	K55.9 - K55.999
	I70.0 - I70.999
	I77.1 - I71.199
	I79.2 - I79.299
Congestive heart failure (CHF)	428.0–428.99
	I50.0 - I50.999
	I09.9 - I09.999
	I11.0 - I11.099
	I13.0 - I13.099
	I13.2 - I13.299
	I25.5 - I25.599
	I42.0 - I42.099
	I42.2 - I42.999
	I43.0 - I43.999
	P29.0 - P29.099
Myocardial infarction	410.0–410.99
	412.0–412.99
	I21.0 - I22.999
	I25.2 - I25.2999
Obesity	278.0–278.999
	E66.0 - E66.999
Diabetes	250.0–250.399
	250.7–250.799
	250.4–250.699
	E10.0 - E10.099
	E10.1 - E10.199
	E10.6 - E10.699
	E10.8 - E10.899
	E10.9 - E10.999
	E10.2 - E10.599
	E10.7 - E10.799
	E11.2 - E11.599
	E11.7 - E11.799
	E12.2 - E12.599
	E12.7 - E12.799
	E13.2 - E13.599
	E13.7 - E13.799
	E14.2 - E14.599
	E14.7 - E14.799
	E11.0 - E11.099
	E11.1 - E11.199
	E11.6 - E11.699
	E11.8 - E11.899
	E11.9 - E11.999
	E12.0 - E12.099
	E12.1 - E12.199
	E12.6 - E12.699
	E12.8 - E12.899
	E12.9 - E12.999
	E13.0 - E13.099
	E13.1 - E13.199
	E13.6 - E13.699
	E13.8 - E13.899
	E13.9 - E13.999
	E14.0 - E14.099
	E14.1 - E14.199
	E14.6 - E14.699
	E14.8 - E14.899
	E14.9 - E14.999
Hypertension	401.9–401.999
	I10.0 - I10.999
Dyslipidemia	272.4–272.499
	E78.5 - E78.599
Ischemic heart diseases	410.0–414.999
	I20.0 - I25.999
Other coronary conditions	429.2–429.299
	I25.10 - I25.099
Stroke	433.01–433.019
	433.1–433.199
	433.11–433.119
	433.21–433.219
	433.31–433.319
	433.81–433.819
	433.91–433.919
	434.00–434.009
	434.01–434.019
	434.1–434.109
	434.11–434.119
	434.91–434.919
	436.0–436.999
	430.0–430.999
	431.0–431.999
	435.8–435.899
	435.9–435.999
	437.3–437.399
	I60.0 - I69.999

### Sample size

The target study sample size is 560 participants allocated equally to the two groups (*n* = 280/group). Sample size estimates were calculated based on the primary outcome of hospital utilization at 30 and 180 days, and the secondary outcome of changes in patient-reported outcomes. All estimates were generated using RMASS2 [47] assuming a statistical significance level of .05 (two-tailed), and targeting at least 80% power. Sample size estimates were adjusted to accommodate expected drop-outs/attrition of 10% between each assessment time point (up to 20% total attrition across 6 months). For the primary aim of examining between-group differences in readmission rates, power analyses were based on published effect sizes [48] for hospital utilization, defined as number of hospital readmissions plus emergency department visits. Specifically, a base rate of 0.37 for the UC group was used, and an incident rate ratio of 0.70 was selected to represent a meaningful decrease in the *Mi Puente* group relative to the UC group. Enrollment ratios were kept equivalent between groups in determining the necessary sample size. Power analyses indicated that a sample size of 558 is needed at baseline to find a statistically significant incidence rate ratio of this magnitude given the base rate with 80% power, and allowing for expected attrition of up to 20% over 6 months. We estimated an effect size of *d* = 0.50 as a clinically significant difference or change in a patient-reported outcome such as quality of life/perceived health [49]. Using the same parameters outlined above, a total of 280 participants is needed to find a statistically significant difference between groups in a patient-reported outcome, with 93% power. Thus, the target baseline sample size of 560 participants is sufficient to detect statistically significant differences of clinically meaningful magnitude across 6 months both in the primary outcome of hospital utilization and in the secondary, patient-reported outcomes.

### Recruitment, screening, and enrollment

Patient eligibility is determined through a multi-step process as shown in Table 2. Recruitment is conducted by trained, bilingual and bicultural research staff. Step 1 (pre-screening) determines demographic and health-related eligibility through the examination of electronic medical records (EMR). The research team collaborated with Scripps Health analysts to develop an automated EMR-based patient identification report that includes a list of all potentially eligible patients (based on Latino ethnicity, age ≥ 18 years, and diagnosis of two or more cardiometabolic health conditions) admitted during the previous 24 h. Once generated, the EMR-derived patient identification report is manually screened by a research assistant to exclude any patients known to be ineligible based on previous involvement with the study (e.g., enrolled in the past 6 months, previously declined participation, exclusionary condition) or based on other EMR information (e.g., cognitive impairment, life expectancy ≤ 6 months, plans to discharge to a SNF). Verified eligible patients are then assigned screening identification numbers for tracking purposes.

**Table 2:** Steps for *Mi Puente* in-hospital screening and consenting

Screening steps	Data source	Screening criteria	Data collected
Step 1 pre-screening	EMR and admission notes	Inclusion: (1) Hispanic ethnicity; (2) ≥ 18 years of age; (3) ≥ 2 cardiometabolic conditions Exclusion: (1) pregnancy; (2) serious life-threatening condition with life expectancy ≤ 6 months; (3) psychiatric morbidity or neurological/cognitive impairment of sufficient severity to preclude consent or participation in the intervention; (4) discharging to location other than home (e.g., SNF); (5) does not speak Spanish or English	Medical information, including previous emergency department admission, chronic condition diagnoses, and LACE index Patient identifying information including name, demographics, contact information, and medical record number
If pass step 1, Step 2 approach in person	Bedside nurse	Patient is available for screening. Yes - approach patient No - e.g., not currently in room or has already been discharged; document reasons and research assistant will return if applicable	New demographic information (e.g., language preference), screening status, qualitative enrollment data to facilitate future approaches/recruitment efforts
If pass step 2, step 3 in-person screening approach	Patient	Confirmation of patient name and language preference. Verbal consent to administer screener No - declined Yes - complete Behavioral Health Screener: ≥ 1 behavioral health concern(s) (i.e., related to mental health, life stressors, medication adherence, healthcare use); telephone access (see Table 3)	Reason(s) for patient eligibility/ineligibility
If pass step 3, step 4 consenting	Patient	Yes – agreed No - declined No - consent not obtained → study was introduced but no decision was made about participation	Complete consent form Reasons for refusals and “hard” refusals (patient explicitly declined enrollment and will not be approached in the future) or “soft” refusals (patient may be approached in a future hospital readmission) Reasons for no decision

*EMR* electronic medical records, *SNF* skilled nursing facility, *LACE* “length of stay”, “acuity of the admission”, “comorbidity of the patient”, and “emergency department use in the 6 months before admission”

Research assistants then consult with hospital staff (step 2) to confirm that each patient is available to be approached and if so, they proceed with step 3 (screening). Research staff confirm that the patient speaks English or Spanish, introduce the study, verify telephone access, and obtain verbal consent to administer the Behavioral Health Screener, shown in Table 3. If eligibility is confirmed and the patient expresses interest in participation, the research assistants proceed with step 4 (informed consent).

**Table 3:** Mi Puente Behavioral Health Screener and Eligibility Determination

Measure	Number of Items	Description	Eligibility Determination
Proactive Health Management			
Medication adherence	1 item	This study-specific item asks patient to indicate the number of days recommended medication doses were missed in the past 7 days.	Missed “sometimes,” “often,” or “always”
Healthcare utilization	5 items	This study-specific measure assesses routine medical care access/use in the past 3 months. Lack of routine medical care is defined as: No routine medical exam, or patient unable to recall date of last routine medical exam; patient not able to receive health care when needed, or; patient endorses uses emergency room or hospital outpatient department for routine medical care.	Lack of routine medical care
Substance Use			
Alcohol	4 items: Alcohol Use Disorders Identification Test-C (AUDIT-C)	This measure screens for risky drinking behaviors based on sex-specific cut scores. Scores ≥ 5 for men (i.e., consuming ≥ 14 drinks per week or ≥ 5 drinks in one occasion ≥ 1 times per month) and ≥ 4 for women (i.e., consuming ≥ 7 drinks per week or ≥ 4 drinks in one occasion ≥ 1 times per month), may be indicative of hazardous drinking. This measure has demonstrated validity in both men and women in primary care settings [50] and has been recommended for use in general health screening [51].	Women: score ≥ 4Men: score ≥ 5
Smoking	1 item	This item assesses if patient currently smokes cigarettes (Yes/No).	Endorses current smoking
Emotional Well-Being			
Anxiety symptom screener	2 items: Generalized Anxiety Dissorder-2 (GAD-2)	This scale assesses the frequency of anxiety symptoms experienced over the past 2 weeks [52].Scores ≥ 3 may be indicative of an anxiety disorder. This measure has demonstrated validity across diverse primary care patients ([52], including Spanish-speakers [53].	Score ≥ 3
Depression symptom screener	2 items: Patient Health Questionnaire (PHQ-2)	This scale assesses the frequency of depressed mood and anhedonia over the past 2 weeks. Scores ≥ 3 may be indicative of clinical depression. This measure has demonstrated validity in primary care [54] and general medical outpatients [55].	Score ≥ 3
Chronic stress	12 items: Chronic Burden Scale	Assesses the number of current ongoing problems of at least 6 months duration in major life domains (i.e., financial, work, relationship, health problems in self or close other, drug or alcohol problems in close other, caregiving, other chronic stressor) [56]. This measure has been used in prior multi-ethnic and Hispanic cohort studies [57, 58], and scores shown to relate to cardiometabolic disorders and risk factors [58–60].	Score ≥ 1 chronic stressor
Chronic health problem distress	2 items	Diabetes Distress Screener [61], adapted to assess distress associated with chronic health problems experienced in the past month. Specifically, the participant felt “overwhelmed by the demands of living with chronic health problems” or felt he/she was “failing with health care regimen.”	Score ≥ 6
Telephone Access			
Telephone access	1 item	Assesses if patient has access to a United States based telephone number that can be used for the duration of the study.	Telephone access endorsed

#### Informed consent

Research assistants review a paper copy of the informed consent document with the patient in his/her preferred language. The patient’s comprehension is monitored and the consent process is halted if a patient demonstrates difficulty understanding the content of the study or the informed consent document. Where questions about ability to consent arise, research assistants consult bedside nurses to determine if the difficulties are temporary or are unlikely to be resolved. If it is determined that a patient will have continued difficulties providing informed consent, the patient is categorized as ineligible. If the patient has difficulties reading, the consent form is read aloud word for word in the presence of a witness (e.g., family member). Once ample opportunity to ask questions about the study has been provided, participants are asked to provide written consent. Reasons for refusal to participate will be recorded where possible.

### Interventions

The two-group parallel design compares UC discharge procedures to the *Mi Puente* specially trained behavioral health nurse plus volunteer community mentor team intervention.

#### Group 1, usual care (UC)

Patients in this group receive the hospital’s UC approach as documented in the discharge instructions. This includes education materials, appointments to see outpatient medical providers (e.g., primary care, cardiology), and referrals to other community-based providers (e.g., home care). The UC condition reflects evidence-based discharge approaches that enhance coordination of care for individuals with complex needs who are at risk of readmission. UC components are individually tailored and provided by hospital inpatient navigators and case management representatives who troubleshoot difficult aspects of discharge planning, including arranging outpatient follow-up appointments and/or diagnostic tests and resolving insurance or billing issues. Patients receiving UC are provided with printed behavioral health services information as appropriate. Patients with acute psychiatric needs are referred to the inpatient psychiatric consultation-liaison team for triage and evaluation.

#### Group 2, *Mi Puente* (My Bridge)

**Fig. 1:**
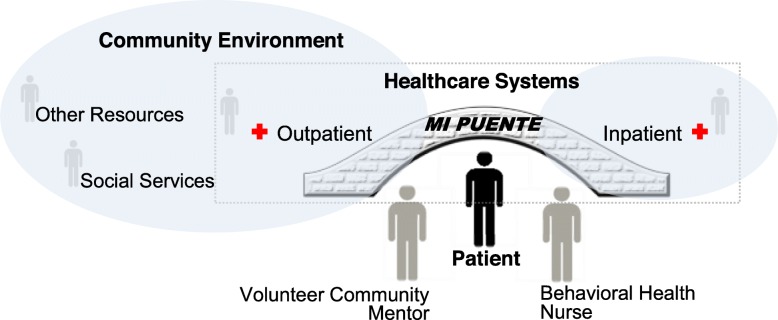
Conceptual overview of *Mi Puente* intervention. Behavioral health nurse and volunteer community mentor provide bridging support, which is enhanced by strong relationships between inpatient facility and community partners in order to achieve reduced hospital utilization and improved patient-reported outcomes

In addition to the processes described for UC, patients assigned to the *Mi Puente* group receive an innovative, team-based intervention to address their integrated health needs during the transition to home. The format and content of the *Mi Puente* intervention were guided by input from inpatient and outpatient care providers and administrators, community organizations, patients, and patient caregivers, collected during the pre-trial formative phase, and using experience gained during a pilot intervention [62]. The building blocks of the *Mi Puente* intervention strategy are a specially trained behavioral health nurse (inpatient “anchor”), a volunteer community mentor (“connecting archway”), and partnerships with outpatient medical facilities (outpatient “anchor”) and community organizations (see Fig. 1). In line with the cultural relevance of interpersonal relationships among Latinos [63] and the importance of involving caregivers and family as key members of the care transition process, support person(s) are included in the intervention whenever possible. The overarching goal of *Mi Puente* is to provide care transition support to patients and their caregivers for up to 30 days following discharge. The specific intervention components are tailored to the patient and caregiver needs and preferences.

**Fig. 2:**
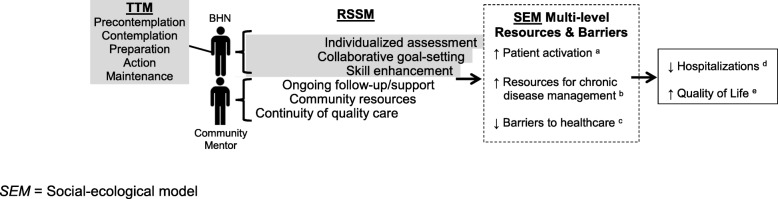
Theoretical mapping of *Mi Puente*. Using the transtheoretical model (TTM) to assess readiness for change, and targeting resources and supports for self-management (RSSM) components 1–3 (behavioral health nurse (BHN)) and 4–6 (volunteer community mentor), the *Mi Puente* intervention will increase resources and decrease barriers across multiple socioecological levels. The operationalization of all RSSM components in intervention content is monitored using behavioral health nurse “Ready, Set, Action” forms and volunteer community mentor checklists (See Additional file 1). Primary outcomes (d, e) and proposed mechanisms (a–c) are operationalized with the following measures: (a) Patient Activation Measure, (b) Chronic Illness Resources Survey, (c) measure adapted from the Hispanic Community Health Study/Study of Latinos, (d) hospital utilization assessed by electronic medical record (EMR) and self-report, (e) Patient-Reported Outcomes Measurement Information System (PROMIS) General Health Scale. *SEM* Social-ecological model

*Mi Puente* is based on three complementary frameworks for effective chronic disease management interventions (see Fig. 2). First, the social ecological model [64, 65] asserts that influences on health and behavior exist across multiple levels of society - i.e., individual, interpersonal, organizational, community, and policy - and that interventions must consider health-related barriers and resources at each of these levels to achieve desired outcomes. *Mi Puente* addresses these multi-level factors through the 6 components of the resources and supports for self-management model (RSSM) [66, 67]: (1) individualized assessment; (2) collaborative goal-setting; (3) skills enhancement; (4) ongoing follow up and support; (5) community resources; and (6) continuity of quality care. These RSSM components are targeted in the interventions delivered by the *Mi Puente* behavioral health nurse and volunteer community mentor. As research has shown that interventions based on the transtheoretical model (TTM) [68, 69] effectively enhance RSSM components 1–3 [70], the TTM is used to guide the behavioral health nurse’s specific intervention strategies. In brief, the TTM posits that “readiness for behavior change” exists on a continuum, ranging from pre-contemplation to action and maintenance, and individuals can move back and forth between these stages over time. By assessing the location of an individual on this spectrum, the behavioral health nurse can “meet the patient where he/she stands” and choose stage-appropriate behavior change tools. Finally, the *Mi Puente* volunteer community mentor addresses RSSM components 4–6 by serving as a trusted bridge, support person, and cultural liaison to promote better links with ambulatory healthcare and community resources.

#### Behavioral health nurse intervention

After enrollment, *Mi Puente* participants are visited in the hospital by a specially trained, bilingual behavioral health nurse. The behavioral health nurse holds a BSN and RN qualification, and has received specific training in cardiometabolic conditions, behavioral health, and related interventions. The behavioral health nurse is supported by, and integrated with the Scripps inpatient advanced practice nurses in diabetes, cardiology, and behavioral health. The behavioral health nurse provides an in-person intervention and follow-up phone support encompassing as many intervention components as deemed necessary and for which time allows. Table 4 provides a detailed description of the intervention components conducted by the behavioral health nurse. The intervention content and materials were informed by previously established evidence-based discharge support programs, including the Coleman care transitions intervention [71–73] and project re-engineered discharge (RED) [48, 74], by our pilot intervention program [62], and by input from patient, healthcare system, and community stakeholders.

**Table 4:** *Mi Puente* intervention components

Interventionist	Intervention component	Content	Rationale	RSSM component	Mode, timing, and frequency of delivery
Behavioral health nurse	Needs assessment	The BHN will gather information from recruiting staff, and review the Study-Specific Patient Report and EMR to complete the Needs Assessment Form. The BHN will also use this information to begin completing the Ready Set Action Plan form, highlighting possible areas for discussion and goal setting during the in-person visit (See Study-Specific Patient Report, Needs Assessment Form, and Ready Set Action Plan in Additional file 1)	To determine the severity and/or underlying causes (e.g., language barriers, health literacy, education, social or financial circumstances) of the patient’s behavioral health concerns. To help the BHN tailor information-seeking, education, action planning, problem-solving and behavioral change techniques	Individualized assessment	Forms completed before and during inpatient visit, with patient (and caregiver if available)
Behavioral health nurse	Create patient-specific personal health record (My Personal Health Record)	The BHN and participant will complete the “My Personal Health Record” (MPHR), a written document containing CM and BHN contact information; reasons and dates of admission and discharge; brief medical history summary (including list of current chronic health diagnoses, most recent laboratory results, and recent vaccinations); primary care provider, specialist, and pharmacy information (i.e., name and contact information, reasons for appointment(s), and questions for the provider); medication log (including previously and newly prescribed prescriptions, purpose, dosage, and timing); follow-up medical appointment calendar; and list of relevant resources (see My Personal Health Record in Additional file 1). The participant is encouraged to take their MPHR to their outpatient appointment/s. The MPHR is also copied and shared with the assigned CM	To help educate patients on their health conditions and self-management. To help patients organize information relevant to their care transition and healthcare, including their personalized action plans and goals, post-discharge medication regimens, and follow-up medical appointments To facilitate interactions with medical providers in future follow-up appointment(s)	Skills enhancement	MPHR completed before and during inpatient visit, with patient (and caregiver if available)
Behavioral health nurse	Engage patient in goal setting and action planning (My Action Plan)	Guided by the TTM, motivational interviewing is used to explore stage of change, motivation, elicit change talk, and empower patients to take goal-oriented action to manage their health. The BHN will utilize the Ready Set Action Plan to guide the patient in formulating goals and creating an action plan shaped by the participant’s individual strengths and the multi-level barriers he/she may experience. Action plan goals will use the evidence-based specific, measurable, attainable, relevant, time-bound (SMART) formulation. The participant will complete a My Action Plan form with his/her stated goals, steps, and confidence level in achieving the stated goal for each domain for which he/she is ready to set goals (see My Action Plan in Additional file 1). All My Action Plans will be photocopied and stored in the participant’s file for future intervention contents. The BHN will reinforce the action plan and SMART goals during the post-discharge telephone call and upon readmission, if relevant	To aid participant in formulating and taking action towards improving self-management for chronic condition(s) in an evidence-based format	Collaborative goal-setting	During inpatient visit with patient (and caregiver if available) During follow-up telephone call/s if necessary. During readmission visit if necessary
Behavioral health nurse	Medication review	The BHN will review the participant’s pre-hospital medication and discharge medication lists and help the participant complete the medication log section of their MPHR, explain refill information, and explore beliefs, barriers, or concerns around medication. The MPHR medication log will include previously and newly prescribed medications, their purpose, dosage, and timing Last, the BHN will emphasize the importance of bringing all medications and the medication log to outpatient medical appointment/s During the follow-up call, the BHN will identify any medications that were prescribed but not obtained, identify medication discrepancies, develop a plan to resolve discrepancies, answer questions about medications, and encourage use of patient’s MPHR medication log	To help patients understand and organize post-discharge medication regimenTo address any barriers or concerns regarding medications To facilitate outpatient appointment efficiency and effectiveness	Skills enhancement	During inpatient visit with patient (and caregiver if available) During follow-up telephone call/s During readmission visit if necessary
Behavioral health nurse	Health education	The BHN will provide participants with a health education handout on proactive and reactive behavior, and will discuss and explain chronic conditions and the need for ongoing self-management (see Living with Chronic Illness Handout in Additional file 1)	To provide education surrounding patient’s current chronic conditions (e.g., mechanisms, rationale behind self-care)	Skills enhancement	During inpatient visit with patient (and caregiver if available) During follow-up telephone call/s if necessary During readmission visit if necessary
Behavioral Health Nurse	Condition red flags	The BHN will discuss how to distinguish between medical emergency situations and when it is appropriate to utilize outpatient care. The BHN will also review steps to take in the case of a medical emergency	To reduce unnecessary emergency service utilization and encourage appropriate use of outpatient care	Skills enhancement	During inpatient visit with patient (and caregiver if available) During follow-up telephone call/s if necessary. During readmission visit if necessary.
Behavioral Health Nurse	Provide referrals	The BHN will confirm which referrals were already provided by hospital staff (e.g., case manager) and assist with any of the following referrals deemed appropriate: condition specific education; nutrition services; outpatient navigator; pharmacist; short-term SNF; social services; wellness center; behavioral health; and substance abuse. If patient is discharged before intervention can be completed, the BHN may also provide a Resource Page containing information on commonly used community resources (see Community Resource Page in Additional file 1). Any additional referrals will be made by assigned CM	To provide patient with additional referrals, not already addressed by the hospital staff	Individualized assessment	During inpatient visit with patient (and caregiver if available) During follow-up telephone call/s if necessary During readmission visit if necessary
Behavioral Health Nurse	Ensure understanding of discharge plan	The BHN will discuss discharge plans with participant (when available) to ensure instructions are well understood	To ensure participant understands necessary action following discharge	Skills enhancement	During in-patient visit with patient (and caregiver if available)
Behavioral Health Nurse	Outpatient appointment coordination	The BHN will help the patient complete the medical records release form for the primary care physician (PCP) and specialist visits, encourage patients to follow through with appointments, help the patient compose questions to ask their PCP or specialists, and role-play appointment scheduling and visit scenarios. To organize outpatient appointments, the BHN will aid the participant in completing the MPHR appointment calendar. The BHN will encourage and assist the participant to complete a medical records release form to expedite the transfer of medical records to the participant’s PCP, specialists, and/or personal address. Participants who cannot complete the form while inpatient will be provided with instructions on what items must be included and where they must submit the completed form (see Medical Records Release Form and Medical Records Release Form Guide in Additional file 1) The BHN will inquire about follow-up appointments and transfer of medical records	To expedite the transfer of medical records to the participant’s PCP, specialists, and/or personal address, and support a proactive approach to healthcare visits; to facilitate more effective and efficient outpatient care	Skills enhancement	During in-patient visit with patient (and caregiver if available) During follow-up telephone call/s if necessary During readmission visit if necessary
Volunteer community mentor	In-person hospital visit	If the BHN and CM schedules align with the participant’s availability, the BHN will provide a “warm hand-off” after they conduct their inpatient visit, introducing the CM to the participant as part of the team. Depending on schedules, the CM may need to conduct an in-person introduction without the BHN present, or may need to meet the patient before the BHN conducts the in-person visit (see CM In-Person Visit Checklist in Additional file 1) During this in-person meeting, the CM and the participant will decide on a time for the first telephone appointment. If a PCP appointment has already been scheduled, an appointment with the CM is set before this appointment and noted on the participant’s MPHR. If a PCP appointment has not yet been scheduled, the first telephone call is scheduled for a time during the first week post-discharge	To reinforce the team-care model, build rapport between the CM and participant, and ensure patient understanding of CM role.	On-going follow up and support	During inpatient visit, with patient (and caregiver if available)
Volunteer community mentor	Support follow-up calls	At minimum, CMs place follow-up calls to patients during post-discharge weeks 1 and 2. Participants who have not completed their outpatient medical appointments, and/or who would benefit from additional support (per the CMs’ discretion), will receive additional calls during post-discharge weeks 3 and 4. For patients who are readmitted to the hospital or sent to a skilled nursing facility (SNF) during this 30-day period, the CM has the flexibility to extend phone support The two primary goals of CM follow-up calls are to (1) foster accountability as the patient makes progress towards his/her goals and (2) help the patient problem-solve around multi-level barriers to implementation (see CM Phone Call Checklist in Additional file 1). To achieve goal 1, CMs utilize skills such as motivational interviewing and active listening to guide conversations about behavior change with patients. For goal 2, CMs utilize a Community Resource Manual to provide participants with information on how/where to get assistance needed (see “referrals” section)	To foster accountability as the patient makes progress towards his/her goals and to help the patient problem-solve and overcome multi-level barriers to implementation	On-going follow up and support	By telephone, once per week for up to 4 weeks post discharge
Volunteer community mentor	Provide referrals (as needed)	The CM will refer patients to local community resources listed within the Resource Manual, depending on individual patient needs. This manual was created and is regularly updated with assistance from the study community partners (the partner FHQC, the Chula Vista Community Collaborative, and the Chula Vista Well-Being Center). The manual contains resources covering the following topics: housing and food security; mental health; transportation; insurance/benefits; emergency services; health education and services related to chronic health conditions (e.g., cancer, HIV)	To provide referrals to outside community agencies and resources that may aid the patient in addressing barriers and health needs	On-going follow up and support	By telephone, once per week for up to 4 weeks post discharge During readmission follow-up visit if necessary
Behavioral health nurse and community mentor	Readmission follow-up visit	The intervention team is provided a list of patients who are currently enrolled in *Mi Puente* and have been readmitted to the hospital on a daily basis. Based on interventionist availability, either the CM or the BHN, or both, will meet with the patient in person. The interventionist will utilize past CM and BHN notes to gather information that may inform the readmission follow-up visit (e.g., content of past follow-up calls, past SMART goals, resources provided). The goal of this visit is to review patient progress and provide additional support and resources as needed (see Re-admit Checklist in Additional file 1.	To provide support to patients who have been readmitted to the hospital during their time in the study	On-going follow up and support	During readmission follow-up visit, with patient (and caregiver if available)

*CM* community mentor, *BHN* behavioral health nurse, *emr* electronic medical records, *pcp* primary care physician, *smart* specific, measurable, achievable, time-bound, *MPHR* My Personal Health Record, *TTM* transtheoretical model, *SNF* skilled nursing facility

Prior to initiating the in-person intervention, the behavioral health nurse conducts a needs assessment to determine the severity and nature of the patient’s behavioral health concerns and important contextual factors (e.g., language barriers, health literacy, education, social or financial circumstances, fatalistic beliefs toward health) and any other information that may guide the post-discharge plan (e.g., hospital discharge orders, medication). The behavioral health nurse communicates with recruitment staff, reviews the *Mi Puente* patient report, which highlights behavioral health concerns identified during the screening and baseline assessment (see Table 4 and Additional file 1), and consults the patient EMR, including case manager notes, to inform the needs assessment. This information is then summarized using the Needs Assessment Form and Ready Set Action Plan (see Table 4 and Additional file 1) and used to tailor education, information-seeking, action planning, problem-solving, and behavior change techniques throughout the intervention.

Optimally, each patient meets with the behavioral health nurse for 30–45 min before they are discharged. Caregivers are encouraged to participate, as desired by the patient. During the in-person visit, the behavioral health nurse reviews and ensures the participant’s understanding of their discharge plan, reviews current medications, helps the patient complete the “My Personal Health Record” (MPHR) for future medical visits, helps the patient create a “My Action Plan” containing one or more specific, measurable, achievable, relevant, and time-bound (SMART) goals, provides health education, highlighting chronic health condition “red flags” and a health education handout (see Additional file 1, “Living with Chronic Illness”) and/or provides referrals. The behavioral health nurse uses the Behavioral Health Nurse Checklist to guide the visit and ensure that all relevant content and materials are covered. At the conclusion of the in-person visit, the behavioral health nurse schedules and explains the purpose of the post-discharge follow-up phone call. If the participant is discharged prior to completion of the in-person intervention, the behavioral health nurse mails intervention documents, a Medical Release Form and Medical Release Form Guide, and a Resource Page containing information on commonly used community resources to the patient’s home address to facilitate intervention completion via phone. These forms can be viewed in Additional file 1.

The behavioral health nurse follow-up call is approximately 30 min in duration and is scheduled to occur before the participant’s outpatient primary care appointment and within 3 days of discharge. All *Mi Puente* participants receive one follow-up call; however, if in-person intervention components were not completed before discharge, additional phone calls may be required. During the follow-up call, the behavioral health nurse asks about the patient’s transition home and recent primary care provider visit. The behavioral health nurse also reinforces any My Action Plan SMART goals, discusses post-hospital discharge medication regimens, and encourages use of the MPHR. The behavioral health nurse answers participants’ clinically relevant questions (e.g., about medications and symptoms) and provides or reinforces health education when relevant. Additional details about the behavioral health nurse follow-up call is provided in Table 4.

After the behavioral health nurse completes his/her follow-up call, he/she may reengage by phone with the participant if a clinical issue (e.g., questions about symptoms or medications) arises during a volunteer community mentor call. If readmitted within 6 months of enrollment, patients are visited by the behavioral health nurse (and/or volunteer community mentor) while hospitalized, when possible (see “Re-admit Checklist” in Additional file 1). During these visits, the intervention team provides a new Medical Release Form, discusses the reason(s) the participant was readmitted, and briefly reviews and reinforces intervention components most relevant to the participant.

#### Volunteer community mentor intervention

Volunteer community mentors are Spanish-English bilingual, bicultural individuals with “lived experience” who reside in, or are familiar with the South San Diego community. Persons with “lived experience” are those who have experienced the condition(s) of interest personally or in others, and have accessed or are familiar with the healthcare system. The volunteer community mentor serves as an advocate and support resource to the patient for up to 30 days post discharge as he/she embarks on his/her journey to better health and well-being. The components of the volunteer community mentor intervention are detailed in Table 4.

Ideally, the patient is introduced to the volunteer community mentor by the behavioral health nurse via a “warm hand off” (personal introduction) in the hospital. The warm hand-off fits with the cultural relevance of interpersonal relationships and personal, face-to-face interactions in the Latino population [63]. The goal of the volunteer community mentor’s in-person visit is to build rapport, explain his/her supportive role, and discuss the plan for telephone follow up after discharge. If the participant has already scheduled a primary care appointment, a phone call with the volunteer community mentor is scheduled before the appointment and noted on the patient’s MPHR. If a primary-care appointment has not yet been scheduled, the first phone call is scheduled during the first week post discharge, in part to ensure that the initial follow-up appointment is made in a timely manner. To enhance communication and transparency, the behavioral health nurse provides both the patient and the community mentor with a copy of the Ready Set Action Plan form with the patient’s chosen action plan(s) and SMART goal(s). Thereafter, the volunteer community mentor fosters accountability as the patient makes progress towards his/her goals and is available to help the patient problem-solve around multi-level barriers to implementation via follow-up phone calls.

At minimum, volunteer community mentors place follow-up calls to patients during post-discharge weeks 1 and 2. Participants who have not completed their post-discharge primary care appointment and/or who would benefit from additional support will receive additional calls during weeks 3 and/or 4. If patients are readmitted to the hospital during this 30-day period or discharged to a SNF, the volunteer community mentor may extend phone support or see the patient in the hospital.

The volunteer community mentors use a Community Resource Manual to guide participants on how to obtain assistance needed, based on areas identified in the behavioral health nurse’s needs assessment. The Community Resource Manual was created with assistance from the study community partners and contains resources in the following areas: housing and food security, behavioral health, transportation, insurance/benefits, emergency services, health education, and services related to chronic health conditions (e.g., cancer, HIV). All information related to patient referrals for services are documented in a secure, web-based, Research Electronic Data Capture (REDCap) database [63].

As noted above, participants who are readmitted to the hospital within 6 months of discharge are visited by the behavioral health nurse and/or volunteer community mentor, if available. The interventionist references past volunteer community mentor and behavioral health nurse notes to inform the readmission follow-up visit. The goal of this visit is to review patient progress and provide additional support and resources, and in turn, prevent additional preventable readmissions.

Due to his/her non-clinical role, the volunteer community mentor does not advise participants on any clinical issues that are raised during calls or visits (e.g., questions about medications, symptoms), but refers them to the behavioral health nurse (or emergency services if urgent), who will then contact the patient for triage and assistance with follow-up care as needed. Patients with severe social, cultural, emotional, financial, and other non-clinical barriers that are outside of the volunteer community mentors’ scope of assistance are referred to the CVWBC. Additional information about the volunteer community mentor intervention content and materials is available in Table 4 and Additional file 1.

### Intervention monitoring, adherence, and withdrawals

#### Behavioral health nurse selection, training, and supervision

The behavioral health nurse holds a BSN and RN qualification and is selected based on specific criteria. He/she is required to be bicultural and bilingual in English and Spanish, currently licensed in the state of CA, and to have at least one year of experience working in an inpatient, hospital setting. Additionally, the candidate should have experience caring for and providing education to patients with chronic health condition(s), such as diabetes mellitus and cardiovascular disease, familiarity with discharge planning, connection with ambulatory care and other community resources, and experience identifying/addressing behavioral health concerns. For the current study, the behavioral health nurse received training and ongoing supervision by the primary investigators, a board-certified endocrinologist (APT), CA-licensed clinical psychologist (LCG), and clinical psychologist (ALF).

Prior to study initiation, the behavioral health nurse received 2-day training in “Principles in Health Coaching” led by a national expert and 1-day training in “Motivational Interviewing” led by a CA-licensed clinical psychologist. The behavioral health nurse also received study-specific training (e.g., on forms, procedures) and completed the Collaborative Institutional Training Initiative (CITI) Protection of Human Subjects and the Society of Behavioral Medicine (SBM) Good Clinical Practice certifications. The behavioral health nurse receives biannual “refresher” trainings consisting of seminars on psychosocial support for patients, and formal system-wide clinical/educational training in cardiometabolic treatment advances led by Scripps Health. Weekly case consultation from licensed clinical psychologists was provided in years 1 and 2 of the study, and tapered to monthly (or more frequently as needed) in subsequent years.

The behavioral health nurse receives ongoing support from the Scripps inpatient advanced practice nurses in diabetes, cardiology, and behavioral health to assist with disease-specific clinical issues. Additionally, the behavioral health nurse has access to Scripps inpatient certified diabetes educators who may assist patients with significant disease-specific knowledge deficits. The behavioral health nurse also attends ongoing role-specific and clinical-care-specific training for continuing medical education units.

#### Volunteer community mentor selection, training and supervision

The research team recruits, screens, and selects the volunteer community mentors according to pre-specified criteria - Latino bilingual individuals with lived experience who reside in, and are familiar with the South San Diego community. All volunteer community mentors become Scripps (unpaid) contractors, which includes receiving general volunteer training and ensuring medical clearance and Health Insurance Portability and Accountability Act (HIPAA) compliance. The research team provides additional training and oversight that is specific to the volunteer community mentor role, which includes 1-day Motivational Interviewing training that teaches interviewing skills to assess stage of change and elicit change talk/behaviors. The volunteer community mentors also complete CITI and SBM Good Clinical Practice certification. Weekly supervision is provided by the project manager and clinical psychology doctoral students, supervised by one of the principle investigators (LCG).

#### Intervention fidelity

Intervention content and “dosage” data for *Mi Puente* are ascertained via the (1) Behavioral Health Nurse Checklist, (2) Ready Set Action Plan forms (intervention) and My Action Plans (goal setting), (3) Volunteer Community Mentor Checklists (i.e., date and duration of call/s, main topics, resources information provided). All fidelity data are collected in a REDCap database and reviewed on a regular basis by supervising research staff, who provide informative feedback to the interventionists on adherence to protocols and areas for improvement (forms can be viewed in Additional file 1).

#### Participant withdrawals

Participants who request to no longer receive the intervention are closed out at that time. These participants are referred to as voluntary withdrawals from the intervention but are still tracked for outcomes via medical records abstraction and telephone interviews if contactable. The intervention is permanently discontinued if the participant dies or requests to be withdrawn from the study completely. These participants are referred to as administrative withdrawals and do not receive further contact.

#### Concomitant interventions

*Mi Puente* is conducted in the context of usual care provided by Scripps Mercy Hospital and any outpatient healthcare encounters. The trial provides adjunct services and all participants continue to receive inpatient and outpatient care as usual. There is no restriction placed on concomitant interventions that may be obtained.

### Outcomes assessments

Details on assessment of primary and secondary outcomes, demographic factors, and other variables are shown in Table 5.

**Table 5:** *Mi Puente* assessments of primary and secondary outcomes, behavioral health concerns, and demographic and social contextual factors

Domain	Description	Time of assessment					Number of items
		Screening (pre-allocation)	Base-line	30 days	90 days	180 days	
Primary outcome							
Hospitalizations	EMR data for hospital utilization		X	X		X	n/a
Secondary outcomes							
Physical symptoms/quality of life	PROMIS Global-10 Health Scale [76]		X		X	X	10
Patient activation	Patient Activation Measure [77, 78]		X		X	X	13
Support resources for disease management	Chronic Illness Resources Survey [67]		X		X	X	13
Healthcare utilization	Health Utilization Questionnaire		X		X	X	12
Healthcare access and barriers	Study-adapted measure		X		X	X	5
Behavioral health concerns							
Medication Adherence	Medication adherence	X					1
Smoking	Smoking status	X					1
Alcohol use	Alcohol screener (AUDIT-C) [51]	X					4
Chronic stress	Chronic Burden Scale [56].	X					12
Health-related distress	Study-adapted Diabetes Distress Screener [61],	X					2
Depression	Patient Health Questionnaire 2-item [54]	X					2
Anxiety	Generalized Anxiety Disorder 2-item [52].	X					2
Demographic and social contextual factors							
Demographic information	Age, sex, race, ethnicity, nativity, language, employment, income, education, marital status, housing		X				11
Social support	Single Item Measure of Social Support [79]		X	X	X		1
Fatalism	Fatalism scale [80]		X	X	X		10
Health literacy	Single Item Literacy Screener [81]		X	X	X		1

*EMR* electronic medical records, *PROMIS* Patient-Reported Outcomes Measurement Information System, *AUDIT-C* Alcohol Use Disorders Identification Test

#### Primary outcome

The primary outcome is hospital utilization (readmissions plus emergency department visits) within the first 30 and 180 days following discharge from the initial, index admission. Informed consent includes HIPAA compliant forms and permission to audit the EMR. Subsequent emergency department visits and hospital readmissions across the 180-day follow-up period are abstracted from electronic health records. Audits will examine 30-day and 180-day rates and other information. It is anticipated that most readmissions will occur at the study setting where the participants are enrolled. However, to ensure that ascertainment of the primary outcome is as complete as possible, hospital utilization is also assessed by self-report during the telephone follow-up interviews conducted at 90 days and 180 days following the baseline assessment. It is anticipated that some hospital visits will be missed, because they do not occur at the primary study setting and participants cannot be reached by phone. However, these missing data will occur in both *Mi Puente* and UC participants, and are therefore unlikely to bias the primary outcome assessment. Analyses will include evaluation of missingness and variables that are associated with missing data in a systematic manner will be controlled for in analyses. The primary outcome of hospital utilization is chosen as a clinically relevant indicator of improved quality of care and patient safety, which maps onto the national goal of reducing readmissions as a means to stem rising healthcare costs [82].

#### Secondary outcomes

Measures of secondary outcomes are collected in the hospital during the baseline assessment, and by telephone at 90 and 180 days after baseline (see Table 5). For measures anchored to a specific timeframe, participants are asked about the past 3 months to allow congruent assessment at baseline, 90 days, and 180 days. Quality of life and perceived physical health are assessed using the Patient-Reported Outcomes Measurement Information System (PROMIS®) Global-10 Health Scale [76]. PROMIS is a National Institutes of Health (NIH) initiative to develop state-of-the-science standardized item banks that offer efficient, flexible, and precise measurement of common patient-reported outcomes. The measures were developed using item response theory, are available in Spanish and English (among other languages), and have shown evidence of reliability (internal consistency), validity (ability to discriminate individuals with/without chronic conditions, construct validity, content validity, factorial validity and invariance across age and gender groups) in numerous studies (e.g., [83–86]). The PROMIS Global-10 consists of 10 items that assess physical health, mental health, social health, pain, fatigue, and perceived quality of life, at the time of the assessment, and which are summarized into two subscale scores assessing general mental and physical health. Scores on the measures are calibrated using a *T*-score metric with the mean of the US general population equal to 50 and standard deviation fixed at 10. The scale is internally consistent, and valid in respect to factor structure and magnitude and direction of association with conceptually relevant constructs [87].

Patient activation is an important intermediate process in improved disease self-management and health outcomes [77, 78]. Further, patient activation is believed to be a critical element in efforts to address disparities in health and healthcare quality [88, 89]. We are administering the 13-item version of the Patient Activation Measure (PAM), which assesses patient knowledge, skill, and confidence in self-management activities. The measure queries patient activation in general at the time of the assessment. The PAM has been shown to have strong psychometric properties, including reliability, content, construct, and criterion validity [77, 78]. The measure was translated into Spanish by a bilingual team of translators for a study of US and foreign-born Latinos and was shown to be reliable in both languages [89].

To assess the relative effectiveness of *Mi Puente* versus UC in helping participants to build and capitalize on available resources, participants complete an abbreviated version of the Chronic Illness Resources Survey (CIRS), a measure of resources and supports for self-management over the past 3 months [67]. The CIRS has good psychometric properties [67], is appropriate for use in Spanish-speaking Latinos [90], and emphasizes the importance of building resources for optimal health across multiple levels of the social-ecological model. Participants complete CIRS subscales measuring support resources received over the past 3 months from the participant (personal support, similar to self-efficacy), family and friends, healthcare providers, and community. In a prior trial with a similar population, this 13-item CIRS version was shown to be internally consistent (α = 0.86), and higher resource scores were related to more effective disease self-management and glycemic control [91].

A study-specific measure is used to assess healthcare utilization in the past 3 months. This measure queries participants’ healthcare behaviors including their use of outpatient services, emergency room or urgent-care visits, inpatient stays, inpatient and outpatient surgeries, home health visits, 911 calls, ambulance travel, use of medical equipment and devices, and use of prescription and non-prescription medications. In addition, participants complete a measure of perceived barriers to accessing healthcare needed in the past 3 months. Barriers include difficulty reaching the service by telephone, difficulty obtaining a timely appointment, waiting too long for an appointment, clinic/service not open when healthcare was needed, lack of transportation, lack of access to an interpreter, inability to take time off work, inability to leave caregiving responsibilities, financial obstacles, or legal concerns.

#### Process evaluation outcomes

The RE-AIM model [45, 92] will be used as a framework to evaluate feasibility, acceptability, sustainability, and dissemination and scaling potential of the *Mi Puente* intervention. The evaluation will also be used to guide program revisions prior to dissemination. An overview of the indicators to be assessed from the RE-AIM guided process evaluation is shown in Table 6.

**Table 6:** RE-AIM guided process evaluation framework

Reach	
a) Examine enrollment rate; compare characteristics of eligible participants who enroll versus those who decline	
b) Examine generalizability by comparing sample demographics with those of the target population	
c) Compare participants who received at least 75% of the intended intervention with those who did not and examine differences between these groups	
d) Record detailed information about reasons for, and time of drop-out; compare participants retained versus lost-to-follow-up to examine reasons for attrition	
Efficacy	
a) Assess improvement in primary and secondary outcomes between baseline and month 6 and examine dose-response association (i.e., whether dosage received relates to changes over time)	
b) Examine unintended negative outcomes	
Adoption	
a) Using semi-structured interviews approach, assess Scripps’ stakeholders’ perceptions of the perceived feasibility and efficacy of intervention strategies	
b) Difficulties with implementation	
c) Satisfaction with the intervention, and	
d) Additional benefits derived	
Implementation	
a) Examine intervention dose and fidelity via checklists completed by behavioral health nurse (Ready, Set, Action forms) and volunteer community mentors (Community Mentors Checklists) for each patient interaction and across the intervention	
b) Assess participants’ engagement in the intervention through brief self-reports evaluating satisfaction with the intervention and number of scheduled calls completed	
c) Assess *Mi Puente* participants’ subjective impressions of the content/format of the intervention and materials, satisfaction with knowledge gained, and challenges/barriers experienced via two focus groups (*n* = 20) to be conducted with participants following their completion of the 6-month study protocol	
d) Assess volunteer community mentors’ self-report of satisfaction and conduct in-depth discussions to examine intervention acceptability, and barriers and enabling factors to program implementation	
Maintenance	
a) Assess number of *Mi Puente* participants involved throughout the study period	
b) Reassess stakeholders’ support for more broadly implementing the intervention	
c) Meet with community partners and other stakeholders to discuss dissemination of findings and intervention	

### Participant timeline

**Fig. 3:**
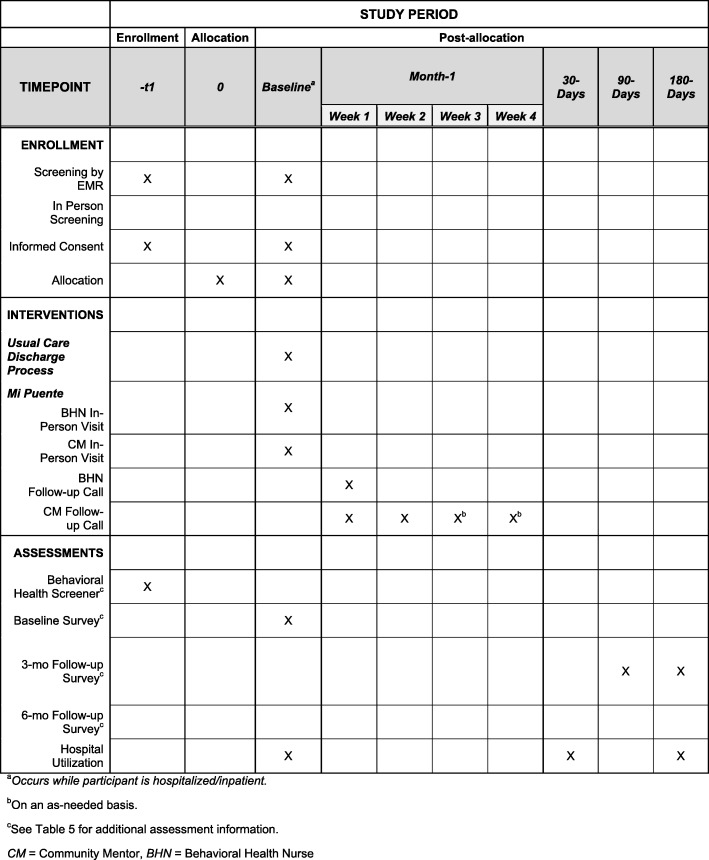
Theoretical mapping of *Mi Puente*. Using the transtheoretical model (TTM) to assess readiness for change, and targeting resources and supports for self-management (RSSM) components 1–3 (behavioral health nurse (BHN)) and 4–6 (volunteer community mentor), the *Mi Puente* intervention will increase resources and decrease barriers across multiple socioecological levels. The operationalization of all RSSM components in intervention content is monitored using behavioral health nurse “Ready, Set, Action” forms and volunteer community mentor checklists (See Additional file 1). Primary outcomes (d, e) and proposed mechanisms (a–c) are operationalized with the following measures: (a) Patient Activation Measure, (b) Chronic Illness Resources Survey, (c) measure adapted from the Hispanic Community Health Study/Study of Latinos, (d) hospital utilization assessed by electronic medical record (EMR) and self-report, (e) Patient-Reported Outcomes Measurement Information System (PROMIS) General Health Scale. *SEM* Social-ecological model

A summary of the expected timeline for participant involvement is shown in Fig. 3. Enrollment occurs during the inpatient hospital stay. After providing written informed consent, the baseline assessment is performed, following which randomization occurs. If randomized to UC, the participant continues to receive inpatient care as usual, followed by UC discharge procedures. For participants assigned to the *Mi Puente* intervention, the behavioral health nurse begins the needs assessment and inpatient component of the intervention as soon as possible. Following his/her visit, whenever possible, the behavioral health nurse introduces the participant to the community mentor via a “warm handoff.” The intervention continues by phone for up to 30 days following discharge, with one call from the behavioral health nurse (within 3 days of discharge) and two to four calls from the volunteer mentor (once per week during weeks 1 and 2 post discharge, and possibly during weeks 3 and/or 4). EMR abstraction is conducted to investigate hospital utilization 30 days post hospital discharge and 180 days post discharge. These follow-up periods were chosen because they are commonly used as indicators of quality of hospital care.

## Methods: assignment of interventions

### Randomization and blinding

The target number of patients to be randomized is 560, equally allocated between the *Mi Puente* and UC conditions (*n* = 280/group). Randomization is unveiled by the research assistant who performs the screening, consenting, and baseline assessment in the hospital, immediately following the baseline assessment. Sequence generation is performed using a computer-generated complete randomization design conducted by the study statistician (SCR), and conveyed to the study project manager. Allocations are indicated by sequential participant number and placed within sealed, opaque envelopes by the study project manager, who is not involved in participant screening, enrollment, or assessment. After the baseline assessment is complete, assignments are opened and revealed by the research staff. Participants and interventionists are not blinded to group assignment, given the nature of the intervention. Research personnel who conduct telephone-based follow-up assessments and EMR abstraction are blinded to group assignment.

## Methods: data collection, management, and analysis

### Data collection methods

Data are collected primarily from participants, and as part of the process aim, from stakeholders such as the behavioral health nurse, volunteer community mentors, community stakeholders, and Scripps staff and administrators. Patient data include hospitalization utilization from the EMR, self-report data collected through in-person and telephone interviews, and qualitative data collected through end-of-study patient focus groups.

#### Electronic medical records abstraction

Baseline clinical data, information on the index hospital admission, and hospital utilization for 180 days following the baseline hospital admission will be abstracted from the EMR by trained research personnel. The data to be abstracted include demographics (e.g., age, race/ethnicity, sex), insurance status, medical history, comorbidities, risk factors, and clinical or laboratory assessments. For each emergency room visit and hospital admission, contextual information such as length of stay, surgeries and procedures needed, medication prescriptions, and admission and discharge diagnosis/es will be collected. The data abstraction report will be developed jointly by study investigators and hospital analysts. To ensure accuracy and completeness of data extraction, two research staff will quality check and verify the extracted data report by spot checking multiple sections of data against live EMR records to evaluate inter-rater reliability. A third staff member will perform the final validation to compile a list of data report feedback to analysts. Analysts will evaluate data discrepancies and troubleshoot extraction procedural issues to produce an updated accurate report. Research staff will perform the quality control-feedback loop until no data discrepancies are found. A REDCap database will be developed to store data abstracted from the EMR for the planned analyses.

#### Patient-reported assessments

The baseline interview is conducted in person while the participant is hospitalized and prior to randomization. Follow-up interviews are conducted by telephone, 90 and 180 days after baseline. All assessments are administered in the participant’s preferred language (English or Spanish), by interview, in order to accommodate the range of literacy levels and health statuses. All interviews are conducted by trained bilingual, bicultural research assistants using a standardized protocol to ensure maximal data quality. Self-report measures were chosen based on evidence of reliability and validity, availability in English and Spanish, and appropriateness to the target population and constructs of interest.

### Data management

All study data are entered into a secure REDCap database, which includes web-based data entry platforms for research staff to enter screening, in-person and telephone-assessment data, and electronic health records abstraction. Study personnel use secure passwords to access the database. Where possible, data fields are preprogrammed to prevent entry of out-of-range or implausible data, and missing data are minimized by requiring that a response is entered before transitioning to the next item. Separate databases are maintained for participant tracking, recruitment, and screening, EMR abstraction, intervention fidelity, and interview/self-report data. The REDCap databases are stored on servers within environments that conform to HIPAA, CITI, and NIH data security regulations and are backed up on a daily basis, with external backups stored off site and exchanged weekly.

### Data quality control procedures

#### Staff training

All research staff are trained and certified in interviewing, questionnaire administration, recruitment procedures, consenting, database use, CITI Protection of Human Subjects, and SBM Good Clinical Practice certification. Research staff also become Scripps contractors, which includes receiving general volunteer training and ensuring medical clearance and HIPAA compliance.

#### Quality control checks

All databases containing study data are checked for completeness and accuracy at least weekly. The number of behavioral health screeners completed and relevant enrollment statuses are cross-checked with the recruitment and screening database to verify accuracy and ensure that coding of patient data is consistent across databases. Baseline, follow-up assessments, and fidelity data are manually checked for completeness and accuracy. Additionally, the number of follow-up surveys completed and appropriate coding of patients (e.g., refusal, deceased) are verified and confirmed. Research staff indicate their name with each survey completed, and are contacted when discrepancies, errors, or omissions of data are identified.

#### Cohort retention procedures

To maximize retention and data quality, participants receive an appointment reminder letter with instructions to contact the study if they need to re-schedule approximately one week prior to follow-up assessments. In addition, approximately 24 h before their appointment, participants receive a reminder phone call. If a participant cannot be reached during three consecutive calls, we attempt to reach alternative contacts if provided. If this does not result in successful contact, public directories are searched. If participants cannot be reached within a month of their 90-day telephone assessment, the data are marked as missing. Participants are then contacted again for their 180-day follow-up assessment, with all retention procedures repeated as outlined above.

### Statistical methods

#### Primary analyses

All analytic strategies will follow published standards, including intent-to-treat principles [93]. Preliminary data screening and cleaning will require examination of distributions for normality, outliers, and missing data patterns at both the univariate and multivariate level. Preliminary inferential statistical testing and effect size consultation will be used to determine if random assignment has resulted in statistical equivalence between groups. Significant covariates will be added to adjust for nonequivalence. Analyses of hospital utilization (primary outcome) and patient-reported outcomes will be conducted using multi-level modeling and the appropriate link function for a target outcome. Multi-level models are especially appropriate for nested data (i.e., time points nested within participants) where missing data and non-normally distributed variables are present [94, 95]. Analyses will include “group” (*Mi Puente* or UC) as the between-subjects factor, “time” (assessments) as the within-subjects factor, and a cross-level, “group-by-time” interaction effect. Follow-up analyses to determine the nature of the differential change between groups will follow recommended procedures [96]. To determine if outcomes differ relative to baseline values, two dummy-coded time variables will be created and specified as level-1 predictors of the target outcome(s) [95]. The baseline assessment will be specified as the referent time point with each follow-up time point, respectively, specified as the comparison time point. Finally, the association between the intervention dosage with the magnitude of change in the target outcomes will be evaluated in the *Mi Puente* group. All analyses will use an intent-to-treat approach, and will be conducted in IBM SPSS Statistics 22.0 (IBM, Inc., Armonk, NY, UK) and MPLUS (Muthen & Muthen, Los Angeles, CA, USA) [97]. Due to the number of statistical tests being conducted, alpha correction will be used to minimize the potential impact of type I error. Effect size indicators and confidence intervals will also be examined and reported. For the primary study aim, additional analyses will be undertaken to fully address the question of interest. Differences in hospital utilization between the *Mi Puente* and UC intervention groups at 30 days and 180 days will be tested using Poisson and proportion tests. Cumulative hazard curves will also be generated and statistically compared using the log-rank test.

#### Cost effectiveness analysis

We will estimate the short-term, within-study cost effectiveness of *Mi Puente* relative to UC from (1) the societal perspective and (2) the healthcare system perspective. Costs will include both the costs of healthcare coordination through *Mi Puente* and the costs of healthcare services received during the study period. Effectiveness will be measured by quality-adjusted life years. The cost effectiveness of *Mi Puente* relative to UC will be estimated using the incremental cost effectiveness ratio, or the difference in costs between the study groups divided by the difference in quality-adjusted life years. The sensitivity of the incremental cost effectiveness ratio to assumptions and estimated parameters will be investigated using a series of one-way and two-way sensitivity analyses [98].

#### Healthcare coordination costs: *Mi Puente*

The *Mi Puente* intervention is conducted by an inpatient behavioral health nurse and a volunteer community mentor. Volunteers are employed in this model in part to promote sustainability and scaling. Costs for the behavioral health nurse will be included in both the societal and health system perspectives, while costs of the volunteer community mentor will be included only in the societal perspective. Time spent by the behavioral health nurse and the volunteer community mentor in supporting study participants will be measured using time-logs tracked in the interventionist checklists (Additional file 1). Time spent by the behavioral health nurse will be valued at the current wage plus benefits, while time spent by volunteer community mentors will be valued at the wage of a community health worker, plus benefits. Overhead, administrative, and phone costs will also be estimated and included in healthcare coordination costs.

#### Healthcare service costs

Costs of healthcare services will be measured using a combination of administrative and self-reported data. Hospital utilization will be measured using administrative data from the participating hospitals (i.e., those in the Scripps system). The resource intensity of each emergency room visit or readmission will be measured using diagnostic related groupings, and the costs will be estimated by applying a national price schedule to these. Non-inpatient services will be measured via telephone follow up at 90 days and 180 days using a self-report assessment of health utilization. This form queries health service use including physician visits, physician phone calls, urgent care and emergency room visits, home health visits, ambulatory surgeries, ambulance transports, and use of prescription medications. Non-inpatient services excepting prescription medications will be identified with their closest corresponding procedure code. The resource intensity of each will be measured according to the relative values units, and the costs will be estimated by applying a national price schedule to the relative values units. Prescription medications will be priced at the average cost for cardiometabolic medications, under the assumption that these are the mostly likely source of any differential medication use among the study groups.

#### Quality-adjusted life years

Quality of life will be measured using the PROMIS global health scale and applying preference weights to responses [86]. The PROMIS provides standardized estimates of well-being from the perspective of patients. The PROMIS items are strongly correlated with quality-of-life measures such as the EuroQoL index, with global PROMIS items accounting for approximately 65% of the variation in EuroQoL scores [86]. We will estimate quality-adjusted life years in this study by applying the coefficients estimated by Revicki et al. [86] to each of the ten global-health item scores.

## Methods: monitoring

### Data monitoring

Barring identifiable problems or substantial risks that would warrant discontinuation of the trial, enrollment will continue until the target sample size of 560 consented and randomized participants is reached. We are actively monitoring progress toward enrollment goals on a monthly basis throughout the recruitment period. Minor modifications were made early in the trial, such as expanding the behavioral health screener (see Table 7), and expanding recruitment coverage, to ensure that enrollment targets are met. We are conducting bi-yearly process evaluations to monitor treatment fidelity and completion rates of key processes including inpatient visits, behavioral health nurse and volunteer community mentor support calls, and completion of 90-day and 180-day patient-reported outcome assessments.

**Table 7:** Major protocol revisions, rationale, and dates

Protocol domain	Protocol revision	Rationale	Date approved
Behavioral Health Screener	Added items to behavioral health screener to increase sensitivity in detecting potential behavioral health issues. Additional items assess healthcare behavior, chronic stress, and chronic disease related distress	We expanded the screener to detect other behavioral health concerns that we felt the original screener was missing, thus increasing the pool of eligible patients who can benefit from the program	The amendment was approved on 10/27/2016
Retention	Began sending a letter to participants in our intervention group when unable to contact for telephone follow up	When unable to contact participants through other means, we send a letter reminding them of available services, and asking them to contact us if desired	The amendment was approved on 10/27/2016
Baseline and follow-up surveys	Housing status item added to baseline and follow-up (3 and 6 month) surveys	Housing and homelessness are important factors that may affect program outcome	The amendment was approved on 1/24/2017
Retention	Began using a public search directory to update phone numbers and contact information when not available from medical records	This change was enacted to maximize participants’ benefit from the intervention, which takes place in part by phone, and to maximize data quality and completeness for outcome assessment	The amendment was approved on 1/24/2017

The study follows a data and safety monitoring plan approved by the funding agency and the Institutional Review Board (IRB). The data and safety monitoring plan includes oversight by a three-member external data and safety monitoring committee. The safety monitoring committee is responsible for safeguarding the interests of study participants, assessing the safety and efficacy of study procedures, reviewing the data, and monitoring the overall conduct of the study. The safety monitoring committee is required to provide recommendations about starting, continuing, and stopping the study. In addition, the safety monitoring committee is asked to make recommendations, as appropriate, about the efficacy of the study intervention; benefit/risk ratio of procedures and participant burden; selection, recruitment, and retention of participants; adherence to protocol requirements; completeness, quality, and analysis of measurements; amendments to the study protocol and consent forms; participant safety; and notification of adverse events. Safety monitoring committee meetings are held yearly and are preceded by the distribution of a report of study progress, adverse events, and other issues of note.

### Harms

The primary study-related risk to participants is the potential loss of confidentiality. Our data management approach includes protections to mitigate this risk. An additional risk is increased distress that could occur as a result of the assessment of behavioral health concerns and/or in response to the intervention. The behavioral health nurse and volunteer community mentors are trained to remain alert to participant distress and provide urgent (e.g., crisis support services, appropriate use of 911 services) and routine psychiatric and medical care referrals (e.g., sources for outpatient healthcare) if needed. All adverse events and other unintended effects of the research and intervention, including loss of confidentiality, are monitored and will be reported to the safety monitoring committee as part of the data and safety monitoring plan.

### Auditing

No outside auditing is conducted as part of the trial.

### Ethics and dissemination

#### Research ethics approval

The research protocol and the informed consent form contained in Additional file 1 have been reviewed and approved by the reviewing IRB (Scripps Health, i.e., the IRB of record performing review on behalf of one or more institutions, also referred to as the single IRB and/or central IRB), with respect to scientific content and compliance with applicable research and human subjects regulations. In addition, all procedures, recruitment, assessment, and intervention materials have been reviewed. All approved documents have been submitted and approved in both English and Spanish language versions. Initial IRB approval was obtained on 29 April 2015. All modifications subsequent to the initial approval have been or will be submitted and approved by the reviewing IRB. The responsible IRBs receive yearly progress reports, including information on the total number of participants enrolled and summaries of each safety and monitoring committee report, and review and approve the study protocol at least annually.

#### Protocol amendments

Any protocol modifications that impact the study conduct, and/or participant risk-benefit profile, including changes in objectives, design, sample size, participant characteristics, staff changes, or significant administrative aspects, require a formal amendment to the protocol. Such amendments are submitted for approval by the relevant IRBs prior to implementation. Minor protocol corrections and/or clarifications that do not affect study conduct or the participant risk/benefit profile are viewed as administrative changes and are documented internally. There have been no protocol changes that would necessitate reporting to the funding agency (i.e., changes that would affect the scope of work or fulfillment of study aims). For a summary of key protocol modifications see Table 7.

#### Informed consent

Initial informed consent is obtained in writing, after review of the study, informed consent form, and ample time to address all questions. The informed consent form is presented in the participant’s preferred language (English or Spanish) by trained bilingual, bicultural research personnel. Informed consent is considered an ongoing process and participants are reminded of the voluntary nature of their participation at each assessment point. The informed consent form has been approved by relevant IRBs, and is shown in Additional file 1.

#### Confidentiality

Participant confidentially is considered of utmost importance by the study investigators. Steps taken to mitigate possible loss of confidentiality include the use of participant identification numbers to label all forms and data, data entry in secure password-protected REDCap data systems, and storage of all hard-copy personal health information in secured, locked file cabinets within offices that operate under strict information security guidelines. The link between participant identification numbers and identity is kept for tracking and follow-up purposes only, and is stored securely and separately from other data. Only trained members of the research team who require access to perform their roles have access to participant identifiers and data collected. All members of the research team are trained to ensure confidentiality and adherence to standardized procedures. All research staff directly involved with the collection and storage of research materials complete the CITI Human Subjects tutorial and the NIH Information Security Awareness Course prior to initiating data collection. Paper copies of data collected are kept in locked cabinets within a locked office. In order to adhere to new NIH data and information security guidelines, cameras are installed in the office where participant printed files are stored and in the server room where databases are stored. All research staff submit a background check prior to being hired for work with the study.

#### Declaration of interests

The study investigators have no financial or other competing interests to declare.

#### Access to data

The study investigators will have full access to and ownership of all data. De-identified data will be made available to interested trainees and outside investigators for additional analyses, upon reasonable request, following reports of primary outcomes, and with appropriate data use agreement.

#### Ancillary and post-trial care

Participants will continue to receive care as usual throughout and following the trial. There is no provision of compensation for harms due to trial participation, and given the nature of the study, harms are not expected.

#### Dissemination policy

To comply with NIH data sharing policies, the study investigators, healthcare and community research partners, and members of the community will develop policies and procedures for sharing data with researchers not affiliated with the original project. We will ensure adherence to all policies and regulations of the Department of Health and Human Service, the NIH, and the participating institutions, Scripps and San Diego State University, including the HIPAA Privacy Rule. We will not directly share qualitative data due to potential for compromising participant identity and related ethical concerns. Broad themes and findings of these data will be shared through publications and presentations. Quantitative written data use agreements will be developed in collaboration with all research partners. Each data use agreement will require that the data be used exclusively for research purposes, for research that entails an inherent benefit to science and society and that includes a comprehensive dissemination plan (to include community and scientific audiences), that no individuals will be identifiable in any manner, that data will be secured using appropriate computer technologies, and that data will be returned or destroyed once analyses are complete. Study findings will be broadly disseminated to the academic/ research community, via journal publications and conference presentations, and to stakeholder (patient, healthcare system) communities, through mechanisms such as lay person or healthcare focused reports, fact sheets, and community presentations. Optimal approaches to dissemination in each context will be developed in collaboration with stakeholder groups.

We will determine authorship using criteria developed by the International Committee of Medical Journal Editors [99]. There is no intention to engage professional writers.

## Discussion

Multimorbidity has reached alarming levels in US adults, and is expected to further increase in prevalence as the population ages and chronic diseases are increasingly diagnosed in younger individuals [100, 101]. The current randomized controlled trial will compare *Mi Puente*, a special care transitions intervention designed to reduce hospital utilization and improve patient-reported outcomes in Latinos with multiple chronic health conditions complicated by behavioral health concerns, to UC, best-practice discharge processes. By conducting the trial at a large safety net hospital that is typical of similar settings across the USA, the study has potential to inform dissemination and scaling of the program if *Mi Puente* is shown to be effective. The *Mi Puente* behavioral health nurse and volunteer community mentor team-based intervention is designed to be cost-efficient, scalable, and to help meet the specific socio-cultural needs of the large and growing US Latino population, while also being adaptable to other conditions and populations. Innovations of the program include the focus on integrated physical-behavioral health care within an inpatient setting, close partnerships with community and outpatient healthcare organizations to ensure maximization of acceptability, feasibility, and uptake, and the use of volunteers as a cost-effective means of broadening the program reach. Importantly, the trial will include a thorough process evaluation and cost-effectiveness analysis. Economic evaluations, and cost-effectiveness studies in particular, in people with multimorbidity will provide critical evidence to inform care models and policies of resource allocation. Identifying resource-efficient interventions that effectively address multimorbidity is one of most important challenges facing our healthcare system today. By introducing the *Mi Puente* care transitions intervention, we seek to contribute to efforts to address the growing and complex healthcare needs of our diverse, high-risk patient populations.

### Trial status

Recruitment started in July 2016 and is ongoing. We expect to complete recruitment in December 2019.

### Protocol version 5 (24 January 2017)

Substantive amendments to the original protocol (approved April 2015) are outlined in Table 7.

## Supplementary information

**Additional file 1. ***Mi Puente* forms and materials.

**Additional file 2.** SPIRIT 2013 checklist.

## Data Availability

The de-identified datasets used and/or analyzed during the current study will be made available from the study investigators following completion of study activities, on reasonable request, and with appropriate data use agreements. Materials not included in this protocol will be made available by request to the study investigators following completion of all study activities.

## Abbreviations

CIRS: Chronic Illness Resources Survey
CITI: Collaborative Institutional Training Initiative
CVCC: Chula Vista Community Collaborative
CVWBC: Chula Vista Well Being Center
EMR: Electronic medical records
FQHC: Federally Qualified Community Health Center
HIPAA: Health Insurance Portability and Accountability Act
IRB: Institutional Review Board
NIH: National Institutes of Health
PROMIS: Patient-Reported Outcomes Measurement Information System
RE-AIM: Reach, efficacy, adoption, implementation, and maintenance
REDCap: Research Electronic Data Capture
RSSM: Resources and support for self-management model
SBM: Society of Behavioral Medicine
SES: Socioeconomic status
SMART: Specific, measurable, achievable, relevant and time-bound
TTM: Transtheoretical model
UC: Usual care
US: United States
