# Supplementary material for: My Bridge (Mi Puente), a care transitions intervention for Hispanics/Latinos with multimorbidity and behavioral health concerns: protocol for a randomized controlled trial
Source: Trials. 2020 Feb 12;21:174. doi: 10.1186/s13063-019-3722-8 (PMC7014646; doi:10.1186/s13063-019-3722-8)
Supplement: Supplementary file 1 — Additional file 1. Mi Puente forms and materials. [file 13063_2019_3722_MOESM1_ESM.pdf]

## Appendix 1: Mi Puente Forms and Materials

- Mi Puente Patient Report
- Needs Assessment Form
- Ready Set Action Plan
- My Personal Health Record
- My Action Plan
- Living with a Chronic Illness
- Behavioral Health Nurse Checklist
- Medical Records Release Form
- Medical Records Release Form Guide
- Resource Page
- Re-Admit Checklist
- Community Mentor In-Person Checklist
- Community Mentor Follow-Up Call Checklist
- Informed Consent

Mi Puente Patient Report

# Behavioral Health Concerns

RA notes for behavioral health nurse:

Expand

Depression

(PHQ-2 score >/=3)

6 View equation

Anxiety

(GAD score >/=3)

6 View equation

Elevated Depression Score

Elevated Anxiety Score

Lack of routine care (months or years)

Experiencing Chronic Stress

Address Chronic Health Problem Distress

## Scores

Patient Activation Raw Score

Level 1 - Disengaged and Overwhelmed: 10-36
Level 2 - Becoming aware, but still struggling: 37-38
Level 3 - Taking action: 39-45
Level 4 - Maintaining and pushing further: 46+

0 View equation

* must provide value

Fatalism Raw Score

Range = 0-10

0 View equation

* must provide value

## For BHN Use Only

Was the BHN meeting successfully completed?

- Yes
- No

reset

BHN Meeting Notes

Expand

Needs Assessment Form

Date Enrolled:
Inpatient visit Date _______________
Time Begin: _________ End: _______
APPOINTMENT
Follow up-Call Actual Discharge date:
Date _________
Follow up call
Date:_____1/5/19_______
Begin: _9:10 A.M._ End: _9:45 A.M.
MI PUENTE Screening # S00000___
PARTICIPANT ID: M000_________
RA: RA Name

Room: *315* Name: *Example Name*
CM: *CM Name*

**Language preference:** SPANISH / ENGLISH

Phone number/s: ___*(619) 000 0000*__
____*(619) 000 0000*______________

Red Cap Completed on: ___________
Added to Intervention List: _________
CE BHN's hotlist: ________________
Consent form in chart:___________

**Behavioral Concerns**:
PHQ-2 + / –
GAD + / -

Tobacco: *Yes*
Alcohol: *No*
Social Support: *Yes*
Chronic **Stress**:
Fatalism: *Yes*

**Difficulties with job/ability to work** –very /mod stressful
**Ongoing financial strain** very /mod stressful
**Helping someone who is sick** very /mod stressful
**Other:**

Lack Preventive Care>3mo: *No*
Med Adherence: *Yes*
Chronic Health Problem **Distress:** *No*
INSURANCE: *Medicare*

**Reason For Adm**:______*Pt. hyperglycemic symptoms*___________________________
**PMH (previous medical hx)**:_________*Dx. T2DM 2012. Most recent admission: 10/5/18 due to hyperglycemicevent.* ___________________________________________
Home Meds: _*Metformin, 500 mg twice a day; ACE inhibitor enalapril (Vasotec), 5 mg daily.*
**WT** __*178 lb*_ **BMI**__*32.6 kg/m2*_ Date:___ *8/23/18*__ Social Hx:_____*Single. Lives alone.*
**BP** __ *150/70 mmHg*_ Date:__ *8/23/18*________
**A1C** ____*8.9*_______ Date:__*8/23/18*______
**Vaccines** __*None*__________ Date: _____
**PCP:** _____ *Dr. Example* ________
Address___________________________________________ Phone_______*(619) 000 000*
__________ FAX________________________________________
**Specialists**___________________________________________________________
______ Specialists______*Dr. Example - endocrinologist*________________________________________
**Allergies**:________*Penicillin*_______________________________________________
Pharmacy Info Address/Phone___*10000 Xst., Chula Vista, CA*______*(619) 000 000* Case Manager:__________*Mr. Example CM*___________________________________
D/C plan Outside services:_____________________________________________

Ready Set Action Plan

| **Ready…**<br><br>As you get **ready** to talk to your patient about an action plan, find out how he/she is currently doing in these areas… | **…Set…**<br><br>Is the patient interested in setting a **SMART** Action Plan? | **Action!**<br><br>Patient's Action Plan |
|---|---|---|
| **_____Depression***<br><br>*If positive PHQ-2, conduct PHQ-9 | _____ Yes<br>_____ No<br>_____N/A | |
| **_____ Anxiety** | _____ Yes<br>_____ No<br>_____N/A | |
| **_____Smoking** | _____ Yes<br>_____ No<br>_____N/A | |
| **_____Alcohol** | _____ Yes<br>_____ No<br>_____N/A | |
| **_____Medication Adherence** | _____ Yes<br>_____ No<br>_____N/A | |
| **_____Routine Visits** | _____ Yes<br>_____ No<br>_____N/A | |
| **_____Social Support** | _____ Yes<br>_____ No<br>_____N/A | |
| **_____Barriers to Healthcare Access** | _____ Yes<br>_____ No | |

Ready Set Action Plan

| | | |
|---|---|---|
| | _____N/A | |
| _____**Chronic Stress** | _____ Yes<br>_____ No<br>_____N/A | |
| _____**Chronic Health Problems** | _____ Yes<br>_____ No<br>_____N/A | |
| _____**Health Literacy**<br>_____**Patient Activation**<br>_____**Housing** | _____ Yes<br>_____ No<br>_____N/A | |

# My Personal Health Record

*(Expediente Personal de Salud)*

________________________________________

Name *(Nombre)*

## *Remember to take this record with you to ALL doctor visits*

**Recuerde llevar este archivo con usted a TODAS las visitas médicas**

This is an **unofficial** medical record provided by the Behavioral Health Nurse to facilitate medical appointments.
Este es un expediente de salud **no oficial** formulado por la Enfermera de Servicios Médicos del Comportamiento para facilitarle sus citas médicas.

# Scripps Mercy Hospital Chula Vista

435 H Street Chula Vista, CA 91910

**(619) 691-7000**

Date of Admission: ___ /___/____ Date of Discharge: ___/___/_____
*(Fecha de admisión)* *(Fecha del día de alta)*

If you have questions or concerns regarding Mi Puente Study, please contact:
*(Si tiene alguna pregunta o preocupación acerca del estudio Mi Puente, por favor de contactar)*

Behavioral Health Nurse: **Name** Phone #: (000) 000-0000
*(Enfermera de Servicios Médicos del Comportamiento)* *(Teléfono)*

Community Mentor:______________________________ Phone #: (000) 000-0000
*(Mentor Comunitario)* *(Teléfono)*

## Reason for Hospital Admission:
*(Razón por Admisión al Hospital)*

## Medical Health History:
*(Archivo de Salud)*

- ☐ Congestive Heart Failure *(Insuficiencia Cardiaca Congestiva)*
- ☐ Diabetes *(Diabetes)*
- ☐ High Cholesterol *(Colesterol Alto)*
- ☐ Stroke *(Derrame cerebral)*
- ☐ Chronic Kidney Disease *(Enfermedad Renal Crónica)*
- ☐ Chronic Obstructive Pulmonary Disease *(Enfermedad Pulmonar Obstructiva Crónica)*
- ☐ Coronary Artery Disease *(Enfermedad de la Arteria Coronaria)*
- ☐ High Blood Pressure *(Alta Presión)*
- ☐ Peripheral Vascular Disease *(Enfermedad Arterial Periférica)*
- ☐ ______________________
- ☐ ______________________
- ☐ ______________________



Mi Puente

# Health Information:
*(Información de Salud)*

## Lab Results
*(Resultados de laboratorio)*

| | | | | |
|---|---|---|---|---|
| A1C | Date *(Fecha)* | | | |
| | Results *(Resultados)* | | | |
| | Date *(Fecha)* | | | |
| | Results *(Resultados)* | | | |
| | Date *(Fecha)* | | | |
| | Results *(Resultados)* | | | |
| | Date *(Fecha)* | | | |
| | Results *(Resultados)* | | | |

| | | | | |
|---|---|---|---|---|
| Weight *(Peso)* | Date *(Fecha)* | | | |
| | Results *(Resultados)* | | | |
| | BMI | | | |
| Blood Pressure *(Presión Sanguínea)* | Date *(Fecha)* | | | |
| | Results *(Resultados)* | | | |

| Vaccinations Received *(Vacunas Recibidas)* | | | | | |
|---|---|---|---|---|---|
| | Flu (Gripe) | Pneumococcal (Neumococo) | Tetanus (Tétano) | | |
| Date *(Fecha)* | | | | | |

# Primary Care Provider Information:

*(Información de Médico de Cabecera)*

Reason for Appointment: **Hospital Follow-up**

*(Razón de la cita:* ***Seguimiento del Hospital)***

Physician's Name: ___________________________

*(Nombre del Médico)*

Phone #:_____________________________

*(Teléfono)*

Address: ___________________________________

*(Dirección)* ___________________________________

___________________________________

Appointment Date and Time: ___________________________

*(Fecha y Hora de la Cita)*

## Questions/Notes for Provider

*(Preguntas/Notas para su médico)*

**Remember to take all medications (prescription and over-the-counter) and hospital discharge paperwork to your appointment.**

*(Recuerde llevar todos sus medicamentos (de receta y sin receta) y todos los documentos recibidos en el hospital a su cita).*



Mi Puente

After your Primary Care Provider refers you to the specialist, write down appointment information here:
*(Después de que su Médico de Cabecera lo refiera al especialista, escriba la información de su cita aquí:)*

# Specialists Information:
(Información de Médicos *Especialistas)*

**1.** Physician's Name *(Nombre del Médico)*: ____________________________ Phone # *(Teléfono)*:______________________________

Address: _____________________________________ Appointment Date and Time: ________________________________________
*(Dirección)* _____________________________________ *(Fecha y Hora de la Cita)*

## Questions/Notes for Specialist:
*(Preguntas/Notas para su especialista)*

**2.** Physician's Name *(Nombre del Médico)*: ____________________________ Phone # *(Teléfono)*:______________________________

Address: _____________________________________ Appointment Date and Time: ________________________________________
*(Dirección)* _____________________________________ *(Fecha y Hora de la Cita)*

## Questions/Notes for Specialist:
*(Preguntas/Notas para su especialista)*

After your Primary Care Provider refers you to the specialist, write down appointment information here:
*(Después de que su Médico de Cabecera lo refiera al especialista, escriba la información de su cita aquí:)*

# Specialists Information:
(Información de Médicos E*specialistas)*

**3.** Physician's Name *(Nombre del Médico)*: ____________________ Phone # *(Teléfono)*:____________________

Address: ____________________ Appointment Date and Time: ____________________
*(Dirección)* ____________________ *(Fecha y Hora de la Cita)*

## Questions/Notes for Specialist:
*(Preguntas/Notas para su especialista)*

**4.** Physician's Name *(Nombre del Médico)*: ____________________ Phone # *(Teléfono)*:____________________

Address: ____________________ Appointment Date and Time: ____________________
*(Dirección)* ____________________ *(Fecha y Hora de la Cita)*

## Questions/Notes for Specialist:
*(Preguntas/Notas para su especialista)*

# Pharmacy Information:
*(Información de la Farmacia)*

Pharmacy Name: ____________________ Phone #: ____________________
*(Nombre de la farmacia)* *(Teléfono)*

Address: ________________________________________
*(Dirección)*

Allergies: ________________________________________
*(Alergías)*

## Questions for the Pharmacists:
*(Preguntas/Notas para el farmacólogo)*

# Medication Log: <u>Morning</u>

*(Registro de Medicamentos: <u>Mañana</u>)*

| **When?** *¿Cuándo?* | **Name/Dosage of Medication/Descriptor** *Nombre/ Dosis del Medicamento/Descripción* | **Why am I taking this?** *¿Por qué tomo esto?* | **New or Old?** *¿Nuevo o Viejo?* | **If "Old"** *Si "Viejo"* | **How Much?** *¿Cuánto?* | **Notes and Reminders** *Notas y Recordatorios* |
|---|---|---|---|---|---|---|
| | **<u>Example</u>: Vitamin D/1000IU/Orange Circle shape** *(Ejemplo: Vitamina D/1000IU/forma redonda y color naranja).* | **General wellness** *(Bienestar general)* | ☑ **New** / *Nuevo* ☐ **Old** / *Viejo* | ☐ Continue ☐ **Stop** / Pare | **1 pill/day** *(1 pastilla x día)* | **I can buy over the counter.** *(La puedo comprar sin receta)* |
| | | | ☐ **New** / *Nuevo* ☐ **Old** / *Viejo* | ☐ Continue ☐ **Stop** / Pare | | |
| | | | ☐ **New** / *Nuevo* ☐ **Old** / *Viejo* | ☐ Continue ☐ **Stop** / Pare | | |
| **Morning** Mañana 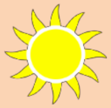 | | | ☐ **New** / *Nuevo* ☐ **Old** / *Viejo* | ☐ Continue ☐ **Stop** / Pare | | |
| | | | ☐ **New** / *Nuevo* ☐ **Old** / *Viejo* | ☐ Continue ☐ **Stop** / Pare | | |
| | | | ☐ **New** / *Nuevo* ☐ **Old** / *Viejo* | ☐ Continue ☐ **Stop** / Pare | | |
| | | | ☐ **New** / *Nuevo* ☐ **Old** / *Viejo* | ☐ Continue ☐ **Stop** / Pare | | |

# Medication Log: Noon

*(Registro de Medicamentos: Mediodía)*

| **When?** *¿Cuándo?* | **Name/Dosage of Medication/Descriptor** *Nombre/ Dosis del Medicamento/Descripción* | **Why am I taking this?** *¿Por qué tomo esto?* | **New or Old?** *¿Nuevo o Viejo?* | **If "Old"** *Si "Viejo"* | **How Much?** *¿Cuánto?* | **Notes and Reminders** *Notas y Recordatorios* |
|---|---|---|---|---|---|---|
| | | | ☐ **New** / *Nuevo*<br>☐ **Old** / *Viejo* | ☐ Continue<br>☐ **Stop** / Pare | | |
| | | | ☐ **New** / *Nuevo*<br>☐ **Old** / *Viejo* | ☐ Continue<br>☐ **Stop** / Pare | | |
| | | | ☐ **New** / *Nuevo*<br>☐ **Old** / *Viejo* | ☐ Continue<br>☐ **Stop** / Pare | | |
| **Noon** Mediodía 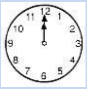 | | | ☐ **New** / *Nuevo*<br>☐ **Old** / *Viejo* | ☐ Continue<br>☐ **Stop** / Pare | | |
| | | | ☐ **New** / *Nuevo*<br>☐ **Old** / *Viejo* | ☐ Continue<br>☐ **Stop** / Pare | | |
| | | | ☐ **New** / *Nuevo*<br>☐ **Old** / *Viejo* | ☐ Continue<br>☐ **Stop** / Pare | | |
| | | | ☐ **New** / *Nuevo*<br>☐ **Old** / *Viejo* | ☐ Continue<br>☐ **Stop** / Pare | | |

# Medication Log: Night

*(Registro de Medicamentos: Noche)*

| **When?** *¿Cuándo?* | **Name/Dosage of Medication/Descriptor** *Nombre/ Dosis del Medicamento/Descripción* | **Why am I taking this?** *¿Por qué tomo esto?* | **New or Old?** *¿Nuevo o Viejo?* | **If "Old"** *Si "Viejo"* | **How Much?** *¿Cuánto?* | **Notes and Reminders** *Notas y Recordatorios* |
|---|---|---|---|---|---|---|
| | | | ☐ **New** / *Nuevo*<br>☐ **Old** / *Viejo* | ☐ Continue<br>☐ **Stop** / Pare | | |
| | | | ☐ **New** / *Nuevo*<br>☐ **Old** / *Viejo* | ☐ Continue<br>☐ **Stop** / Pare | | |
| | | | ☐ **New** / *Nuevo*<br>☐ **Old** / *Viejo* | ☐ Continue<br>☐ **Stop** / Pare | | |
| **Night**<br>Noche | | | ☐ **New** / *Nuevo*<br>☐ **Old** / *Viejo* | ☐ Continue<br>☐ **Stop** / Pare | | |
| | | | ☐ **New** / *Nuevo*<br>☐ **Old** / *Viejo* | ☐ Continue<br>☐ **Stop** / Pare | | |
| | | | ☐ **New** / *Nuevo*<br>☐ **Old** / *Viejo* | ☐ Continue<br>☐ **Stop** / Pare | | |
| | | | ☐ **New** / *Nuevo*<br>☐ **Old** / *Viejo* | ☐ Continue<br>☐ **Stop** / Pare | | |

# Appointment Calendar

*(Calendario de citas)*

## February 2018

*(Febrero 2018)*

| Sunday | Monday | Tuesday | Wednesday | Thursday | Friday | Saturday |
|---|---|---|---|---|---|---|
| | | | | 1 | 2 | 3 |
| 4 | 5 | 6 | 7 | 8 | 9 | 10 |
| 11 | 12 | 13 | 14 | 15 | 16 | 17 |
| 18 | 19 | 20 | 21 | 22 | 23 | 24 |
| 25 | 26 | 27 | 28 | | | |

# Appointment Calendar

*(Calendario de citas)*

## March 2018

*(Marzo 2018)*

| Sunday | Monday | Tuesday | Wednesday | Thursday | Friday | Saturday |
|---|---|---|---|---|---|---|
| | | | | 1 | 2 | 3 |
| 4 | 5 | 6 | 7 | 8 | 9 | 10 |
| 11 | 12 | 13 | 14 | 15 | 16 | 17 |
| 18 | 19 | 20 | 21 | 22 | 23 | 24 |
| 25 | 26 | 27 | 28 | 29 | 30 | 31 |

# Resources

*(Recursos)*

**Participant ID: ___________**

# *My Action Plan*

**Actions:**

☑ Schedule appointment

☑ Take medications

□ Learn more about my disease

□ Apply/Obtain Permanent Insurance

□ Seek Support

□ Reduce/Quit Smoking

□ Reduce/Quit Drinking

□ Other: __________________

**In order to reach my plan**

**I will…**____________________________________________________________________

____________________________________________________________________________

____________________________________________________________________________

**My Confidence Level:**

| **0** | **1** | **2** | **3** | **4** | **5** |
|---|---|---|---|---|---|
| **Not at all Confident** | | | | | **Extremely Confident** |

**SMART** Action Plan = **S**pecific, **M**easurable, **A**ttainable, **R**elevant, **T**ime-Bound

**Participant ID: ___________**

# *Mi Plan de Acción*

**Acciones:**

☑ Hacer cita

□ Buscar apoyo

☑ Tomar medicamentos

□ Reducir/Dejar de fumar

□ Aprender más sobre mi enfermedad

□ Reducir/Dejar de tomar

□ Solicitar/Obtener Seguro Médico

□ Otro: _______________

**Para poder cumplir con mi plan**

**Yo voy a…**______________________________________________

____________________________________________________

____________________________________________________

**Nivel de confianza en mí mismo:**

| | | | | | |
|---|---|---|---|---|---|
| **0** | **1** | **2** | **3** | **4** | **5** |
| **Nada de Confianza** | | | | | **Mucha Confianza** |

Plan de Acción **SMART**= E**s**pecifica, **M**edible, **A**lcanzable, **R**elevante, Limitada de **T**iempo

# Living With a Chronic Illness

*Living with a chronic illness means having a long term condition that may not have a cure.*

Every individual copes with chronic illness differently. While it is normal to feel overwhelmed and helpless, it is important to keep your physical health and emotional well-being as top priorities.

## *Taking Action: Proactive vs Reactive*

Taking care of your health can be done **proactively** or reactively.

**Proactive** means taking your medications as prescribed, and going to the doctor to *stay* healthy. It also means understanding your condition and recognizing early symptoms before they turn into a medical emergency.

Reactive means only getting help when you do not feel well.

## *Emotional Well-Being*

Realize that you are not alone. It is okay to ask for help. Life is busy – it can be overwhelming to keep up with your appointments, medications, and self-care tasks. Whether it be a family member or a health provider, a helping hand can make the world of a difference.

This person can act as support system while making important lifestyle changes such as: changing your diet, exercising, quitting smoking, adjusting to medications, and managing stress.

Finally, be your own best friend! Reward yourself for your successes in managing your health.

## *Find Support!*

It is good to know that you aren't the only one dealing with a chronic illness. You can find support groups online or you can talk with your doctor about local groups. Also, bringing a friend or family member to your support group can help them learn more about your illness.

This is Phil!
Phil is **reactive** when it comes to managing his chronic illness. He:

- Often forgets to take medications.
- Doesn't make changes to his diet and exercise.
- Only goes to the doctor when he feels sick.
- Doesn't reach out to a support system and deals with stress alone.

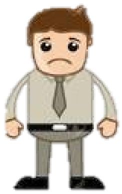

This is John!
John is **proactive** when it comes to managing his chronic illness. He:

- Takes medications as prescribed.
- Includes healthier food options and fits in exercise when he can.
- Goes to the doctor for regular check-ups.
- Reaches out to his support system when he feels stressed.

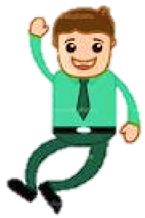

Behavioral Health Nurse Checklist

BHN: Name CM: _________________ M _________ S__________

Was any part of the intervention done in-person (Circle one)? Yes / No SNF (Circle one)? Yes / No

| **Modality (I=In Person P=Phone)** | **Date** | **Total Time** | **Support Person? (Yes/ No)** |
|---|---|---|---|
| ( ) I ( ) P | | | ( ) Yes ( ) No |
| ( ) I ( ) P | | | ( ) Yes ( ) No |
| ( ) I ( ) P | | | ( ) Yes ( ) No |
| ( ) I ( ) P | | | ( ) Yes ( ) No |
| ( ) I ( ) P | | | ( ) Yes ( ) No |
| ( ) I ( ) P | | | ( ) Yes ( ) No |
| ( ) I ( ) P | | | ( ) Yes ( ) No |
| ( ) I ( ) P | | | ( ) Yes ( ) No |
| ( ) I ( ) P | | | ( ) Yes ( ) No |

Behavioral Health Nurse Checklist

| | | | |
|---|---|---|---|
| ( ) I ( ) P | | | ( ) Yes ( ) No |

| Completed at Visit? I | P | Medication | Completed at Visit? I | P | My Personal Health Record (MPHR) | Completed at Visit? I | P | Follow-Up Appointment | Completed at Visit? I | P | Action Plan |
|---|---|---|---|---|---|---|---|---|---|---|---|
| | | Compare pre-hospital medication list with discharge medication with patient | | | Collaborate with patient to complete MPHR | | | Help patient complete Medical Record Release Form for PCP and specialist visits | | | Ready, Set, Action form |
| | | Emphasize importance of taking all medications to PCP appointment | | | Provide patient with copy of MPHR prior to discharge. | | | Encourage patient to follow through with appointment | | | Collaborate with patient complete My Action Plan form |
| | | Discuss medication log in My Personal Health Record | | | Clarify MPHR will be shared with Community Mentor | | | Help patient write questions to ask their PCP or specialist | | | Provide patient with My Action Plan form |
| | | Explain refill information | | | Encourage updating MPHR after PCP/specialist visits | | | Role-play appointment scheduling and visit | | | Reinforce SMART Action Plan post-discharge |
| | | Explore beliefs/concerns around medication | | | Reinforce follow-through with resources provided on MPHR | | | Inquire about recent PCP visit or encourage attending visit (if in future) | | | |
| | | Identify medications that were prescribed but not obtained | | | | | | Inquire if PCP received Hospital Medical Record | | | |
| | | Identify medication discrepancies | | | | | | Inquire about whether patient called to schedule specialist appointment | | | |
| | | Develop a plan to resolve discrepancies | | | | | | | | | |
| | | Answer questions about medications | | | | | | | | | |

Behavioral Health Nurse Checklist

| | | | | | | | | | | | |
|---|---|---|---|---|---|---|---|---|---|---|---|
| | | Encourage use of patient's medication log | | | | | | | | | |

| Completed at Visit? I | P | Condition Red Flags | Completed at Visit? I | P | Referrals by Hospital | Completed at Visit? I | P | Referrals by BHN | Completed at Visit? I | P | Health Education |
|---|---|---|---|---|---|---|---|---|---|---|---|
| | | Was patient asked if nurse provided them with information on medical emergency situations? | | | Case Management ______ ______ ______ | | | Case Management ______ ______ ______ | | | Provide handout |
| | | What to do in case of emergency | | | Condition Specific education ______ ______ | | | Condition Specific education ______ ______ | | | Discuss/explain chronic conditions & need for ongoing self-management |
| | | Discuss when PCP should be called | | | Nutrition Services ______ | | | Nutrition Services ______ | | | Answer Questions |
| | | | | | Outpatient Navigator | | | Outpatient Navigator | | | |
| | | | | | Pharmacist | | | Pharmacist | | | |
| | | | | | Short Term SNF | | | | | | |
| | | | | | Social Services ______ ______ | | | Social Services ______ ______ | | | |
| | | | | | Wellness Center ______ ______ | | | Wellness Center ______ ______ | | | |
| | | | | | Behavioral Health | | | Behavioral Health | | | |

Behavioral Health Nurse Checklist

| | | | | | | | | | | | |
|---|---|---|---|---|---|---|---|---|---|---|---|
| | | | | | Substance Abuse | | | Substance Abuse | | | |

**Date _______/_______/______** **Provided Green Folder? ________________** **Going to SNF (Circle one)? Yes / No**

**In-Person Visit Comments:**

**Behavioral health concerns:**

y/o admitted for. History of Medical Insurance:

**Social History:**

**Current Challenges per pt:**

**Pt enjoys:**

**Pt signed form for RELEASE of MEDICAL RECORDS** for a copy to be sent to Self and PCP

(Dr. ) **Transportation to appointment/**s will be provided by __.

BHN phone call appointment made for:

---

**Reminders for follow-up call:** Action Plan f/up
PCP f/up
Specialist F/up
Pharmacy med pick up
Med Reconciliation
Transportation arrangements Remind CM f/up appt

**Was the PHQ-9 administered? Y / N**
**If yes, what was the outcome:**
**Score:**
**Suicide ideation:**
**Comments:**

**Follow-up call: Date _______/_______/______ Time: ________________ AM PM**

**Follow up Call Comments:**
**DISCHARGE DATE from Hospital :**
**DISCHARGE DATE from SNF :**
**Action Plan f/up :**
**PCP f/up :**
**Pharmacy med pick up :**
**Med reconciliation :**
**Transportation arrangements :**
**Remind CM f/up apt :**

## BHN to CM Communication

| Services Participant is Currently Receiving | Services Pending Upon Discharge |
| --- | --- |
| ▪<br>▪ | ☐<br>☐<br>☐<br>☐<br>☐<br>☐ |
| **Other Comments** | **BHN Recommendations** |
| **Language Preference : English / Spanish**<br>**Address:**<br><br>**Contact numbers:**<br>▪ **(619) (cell)**<br>▪ **(619)**<br>▪ | ☐<br>☐<br>☐<br>☐ |

Medical Records Release Form

Scripps

| *MRN: ____________________ |
|---|
| Facility Use Only |

## Request to Obtain a Copy or Authorization for the Use or Disclosure of Health Information (Medical Records)

*Patient Name: ______________________ Also known as: ____________________

*Address: ____________________________________________

*Date Of Birth: ____/____/____ *Telephone: ( ) ____________________

***Record Holder:**

☐ Scripps Clinic / Coastal Medical Center

☒ Scripps Hospital / Emergency Room Name (enter here): Chula Vista

☐ Scripps Home Health / Hospice

Other: ____________________________________________

***Date of Service:** From ____/____/____ To ____/____/____

***Location of Treatment:** ☐ Inpatient ☐ Emergency ☐ Outpatient ☐ Urgent Care

***Release Records to:** ____________________________________________

____________________________________________

Street Address City State Zip

( ) ____________________ ( ) ____________________

Phone Fax

***I would like the Health Information:** ☐ Paper/Mailed ☐ Electronic/Emailed

Email address: ____________________________________________

I would like my information emailed in a ☐ secure or ☐ unsecured manner (check one)

I acknowledge that by electing to receive my health information via email in an UNSECURED manner, that the information will not be encrypted, and that it could be intercepted and viewed by a third party. Scripps is not responsible for unauthorized access of your health information while in transmission to the email address you designated above.

***Type Of Information:** This authorization is limited to the following medical records and type of information:

☐ Discharge Summary
☐ History/Physical Exam
☐ Consultation Reports
☐ Operative/Procedure Reports
☐ Emergency Department Reports
☐ Office Notes
☐ Laboratory Tests
☐ PT/OT/Speech Therapy Notes
☐ Radiology Images ☐ Radiology Reports
☐ Urgent Care Reports ☐ Entire Record
☐ Other (please specify): ____________________
☐ **HIV (Human Immunodeficiency Virus) test results**
☐ **Mental Health**
☐ **Alcohol and/or Drug Abuse Program Treatment**

***Use of Information:** The requestor may use the medical records and type of information authorized only for the following purposes:

☐ Continuing Care ☐ Legal ☐ Personal ☐ Insurance Claim

☐ Other (Please specify): ____________________________________________

***Printed Name:** ____________________________________________

***Signature:** ______________________________ ***Date:** ______________

If signed by other than patient, indicate relationship: ____________________

Witness: ______________________________

I hereby authorize release of all information as stated above:

**Attending Physician (if appropriate):** ____________________________________________

MD Signature: ______________________________ Date: ______________

*3ROI*



*ROI 100-8700-739SW*

[illegible]

# Medical Records Release Form

The section you need to complete looks like this: (Provide Primary Care Provider information)

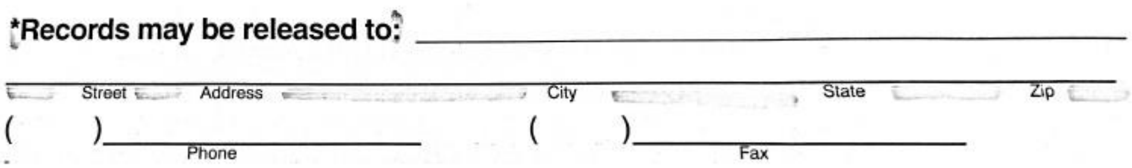
*Records may be released to: ____________________

Street Address City State Zip

( )__________ Phone ( )__________ Fax

- ➔ When you have the entire form completed, visit the Medical Records Office located in Scripps Mercy Chula Vista Hospital, 435 H Street, Chula Vista, 91910
  The office is located in front of Benny Bean's Coffee Cart (near the east entrance)
- ➔ Tel 619-691-7336
- ➔ Fax 619-691-7413
- ➔ <u>Hours of Operation: Monday-Friday 8:30AM-4 PM</u>
  <u>Saturday, Sunday, Holidays: CLOSED</u>

# Divulgación de Archivo Medico

La sección que tiene que completar se ve igual a esto: (Proveer Información de Médico de Cabecera)

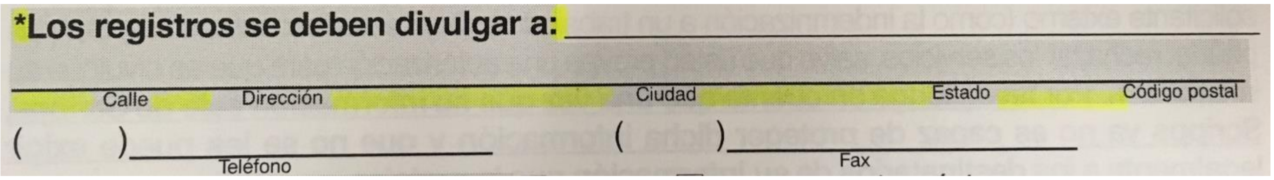
*Los registros se deben divulgar a: ____________________

Calle Dirección Ciudad Estado Código postal

( )__________ Teléfono ( )__________ Fax

- ➔ Cuando tenga todo el formulario completado, visite la Oficina de Registros Médicos situado en el Hospital Scripps Mercy Chula Vista, 435 H Street, Chula Vista, 91910
  La oficina está situada en frente de Benny Bean's Coffee Cart (cerca de la entrada del este)
- ➔ Tel 619-691-7336
- ➔ Fax 619-691-7413
- ➔ <u>Horario: Lunes a Viernes 8AM-4:00 PM</u>
  <u>Sábado, Domingo y Días Festivos</u>
  <u>CERRADO</u>

Scripps

# Patient Community Resources

**Scripps Mercy Hospital Chula Vista**

Medical records:

(619) 691-7336

Physician Referrals:

1-800-727-4777

**Substance Abuse/Mental Health**

Smoking Cessation Hotline

1-800-NO-BUTTS

Mental Health Crisis Line

1-888-724-7240

Alcoholics Anonymous

(619) 476-0288

SMART Recovery (AA alternative)

smartrecoverysd.org

**Mental Health Support Groups**

**NAMI, Chula Vista**

Family Support Groups

(619) 288-3133

*Call about support groups for families

Peer Support Groups

(619) 420-8603

*Call about peer support groups

**Scripps Mercy Chula Vista Well Being Center**

(619) 862-6611

*Call about support groups for those living with chronic illness such as diabetes and hypertension

**Scripps Diabetes Center Chula Vista**

(858) 678-7050

Diabetes center open M, W &F

From 8 am-5pm

*offers a wide variety of services such as individual appointments, group classes, nutritional counseling, and support groups

**Housing**

Scripps Mercy Chula Vista Well Being Center

(619) 862-6602

*Please call for more information regarding help for homeless individuals

**Community Social Services Hotline**

211

*Call for financial, legal, and health assistance. Includes help applying for SNAP and other social benefits.

**Transportation Services**

FACT: Facilitating Access to Coordinated Transportation

(888) 924-3228

*Call for more information about free or low cost transportation services

**Meals on Wheels**

(619) 420-2782

*Delivers fresh meals to seniors and people living with disabilities.

**Pharmacy Offering Home Delivery**

Pride Pharmacy

(619) 501-5888

Monday-Thursday 9 am – 6 pm

Scripps

# Recursos comunitarios para pacientes

**Scripps Mercy Hospital Chula Vista**

Registros médicos:

(619) 691-7336

Referencias de médicos:

1-800-727-4777

**Abuso de sustancias/salud mental:**

Dejar de fumar:

1-800-NO-FUME

Linea de cris de salud mental:

1-888-724-7240

Alcohólicos Anónimos:

(619) 476-0288

SMART Recovery* (alternativa AA)

Smartrecovery.org/espanol/

**Grupos de apoyo de salud mental**

**NAMI, Chula Vista**

Grupos de apoyo para familias

(619) 288-3133

*Llame para más informes

Grupos de apoyo para individuales

(619) 420-8603

*Llame para más informes

**Scripps Mercy Chula Vista Well Being Center**

(619) 862-6611

*Llame para informes sobre grupos de apoyo para gente con enfermedades crónicas como diabetes y hipertensión

**Scripps Diabetes Center, Chula Vista**

(858) 678-7050

*Centro de diabetes abierto L, M &V

8 am - 5 pm

*Ofrece una variedad de servicios como citas individuales, clases en grupo, asesoramiento nutricional y grupos de apoyo

**Alojamiento**

Chula Vista Well Being Center

(619) 862-6602

*Llame para más informes sobre ayuda para gente sin hogar

**Línea directa de servicios de la comunidad:**

211

*Llame para más informes sobre asistencia financiera, legal, o de salud. Incluye ayuda aplicando para SNAP y otros apoyos sociales.

**Servicios de transportación**

**FACT: Facilitating Access to Coordinated Transportation**

(888) 924-3228

*Llame para más informes sobre opciones gratuitas o de precio reducido para adquirir servicios de transporte

**Meals on Wheels**

(619) 420-2782

*Comida saludable a domicilio para gente de la tercera edad y gente discapacitada.

**Farmacia con entrega a domicilio**

Pride Pharmacy

(619) 501-5888

Lunes a jueves 9 am –6 pm

Sábado 9 am– 1 pm

# Re-admit Checklist

| | |
|---|---|
| **M ID / Screening ID** | M________ S________ |
| **Date** | ____ / ____ / ____ |
| **Total Visit Time** | ____:____ |
| **Was a support person involved in this contact?** | ☐ Yes ☐ No |
| **Will patient be going to a SNF?** | ☐ Yes ☐ No |
| **Topics Covered** (select all that apply) | ☐ Medication<br>☐ Follow-Up Appointments<br>☐ Action Plan (check if new action plan created or old action plan reviewed)<br>☐ Condition Red Flags<br>☐ Health Education<br>☐ Referrals by Hospital: ___________________________<br>☐ Referrals by BHN: ______________________________<br>☐ Other: ________________________________________ |
| **In-Person Visit Comments/ Notes** | |

Community Mentor In-Person Checklist

**Before leaving for Scripps:**

| **Patient Specific Information:** | | Mi Puente PID: S__________ M: __________ |
|---|---|---|
| Participant Name: | | Preferred language: Sp / En |
| Nurse: | Nurse's #: | CM Name: |
| Room number: | Start time: | RA Name: |
| Bed Number: | End time: | ☐ RA & CM are the same person |

1. Obtain patient specific information from RA.
2. Ask RA if patient will be discharged any time soon and if it's now a good time to visit. If yes, attempt in-person visit as soon as possible. Date of discharge, if known:_________________________________

**During In-person Visit:**

3. Verify with patient if they have a follow up appointment set and a discharge date. If so, when and where?_____________________________________________________________________________
4. Schedule first call before their PCP appointment or within first week post-discharge, which ever comes first: _______________________________________________________________________________
5. If neither are known, wait for BHN instructions to schedule follow up call.

**After In-person Visit:**

6. Send an email to the Mi Puente Google Group to let everyone know that you have met with the participant, and update REDCap and data entry log.

**If in-person visit is attempted on a different day as day of recruitment, before leaving for Scripps:**

Call patient's nurse to make sure it's OK to visit in the next few minutes for a quick introduction and to verify that the patient is still in the same bed and room number.

If not OK, when is a better time?

_______________________________________________________________________________

**Comments:**

| |
|---|
| |

Community Mentor Follow-Up Call Checklist

CM: ________________________Participant ID:________________________________

# <u>Community Mentor Follow-Up Checklist</u>

| **Completed during call?** | | | | **Transition Home** | **Completed during call?** | | | | **PCP Appointment** | **Completed during call?** | | | | **MPHR** | **Completed during call?** | | | | **Action plan created with BHN** | **Completed during call?** | | | | **Referrals** |
|---|---|---|---|---|---|---|---|---|---|---|---|---|---|---|---|---|---|---|---|---|---|---|---|---|
| **1** | **2** | **3** | **4** | | **1** | **2** | **3** | **4** | | **1** | **2** | **3** | **4** | | **1** | **2** | **3** | **4** | | **1** | **2** | **3** | **4** | |
| | | | | Discuss the transition home | | | | | Ask if the appointment has been scheduled | | | | | Discuss any updates made by patient | | | | | Discuss how the patient is following through with the action plan | | | | | Refer patients to additional services that BHN recommends |
| | | | | Ask if medication was retrieved from pharmacy | | | | | Discuss how the appointment went/discuss the importance of making an appointment | | | | | Ask if the MPHR was used at PCP appointment | | | | | Discuss any possible barriers to following through with the action plan | | | | | Refer patient to Chula Vista Well-Being Center |
| | | | | Ask if any new concerns have arisen post discharge | | | | | Discuss any barrier related to accessing primary outpatient care | | | | | Discuss any updates made by PCP | | | | | | | | | | Refer patients to additional referrals that CM recommends |
| | | | | Ask open ended questions for a better understanding of patients' current state | | | | | Remind patient to take medications and MPHR to their appointment | | | | | Ask if the MPHR was helpful during the appointment | | | | | | | | | | Ask patient about progress on prior referrals/ Discuss barriers to access referrals |

# *Mi Puente*: My Bridge to Better Cardiometabolic Health and Well-Being

## Scripps Health & San Diego State University

## INFORMED CONSENT

Principal Investigators:
Athena Philis-Tsimikas, MD and Linda Gallo, PhD

Co-Investigators:
Addie Fortmann, PhD, Laura Talavera, MSN, MPH, Scott Roesch, PhD, Greg Talavera, MD

Project Managers:
Duvia Ledesma, MA (619) 240-7841 during office hours
Johanna Euyoque, MA (858) 465-9142 during office hours

Research Sites:
Scripps Mercy Hospital, Chula Vista: 435 H Street, Chula Vista, CA 91910
Scripps Whittier Diabetes Institute: 10140 Campus Point Drive, Suite 200, San Diego, CA 92121 San Diego State University: 780 Bay Boulevard, Suite 200, Chula Vista, CA 91910

Sponsor:
National Institutes of Health/National Institutes of Nursing Research (NIH/NINR)

We are asking you to be a part of the *Mi Puente* study. *Mi Puente* is a clinical trial, a type of health research study. Clinical trials include only patients who choose to take part. Before you give your consent to volunteer, please take your time to make your decision. It is important that you read the following information and ask as many questions as necessary to be sure you understand what you will be asked to do.

*Before you start reading about this research, please read the California Experimental Subjects' Bill of Rights, which is page 9 of this form.*

This study is being done by Scripps Health and San Diego State University.

**Why is this research study being done?**
This research study will try a new way to discharge patients from the hospital to their home. Its purpose is:

- To test how well this new procedure works to keep patients from being readmitted to the hospital or visiting the Emergency Department and

- To see if we can improve the way patients feel physically and emotionally.

We will invite 560 patients to join this study over five years. Patients must be hospitalized at Scripps Mercy Hospital in Chula Vista and have certain conditions, which are described below.

**Who is eligible to participate?**
You can participate in this research study if you:

- Consider yourself Hispanic/Latino (and/or Mexican, or Chicano), of any race
- Are in the hospital as a patient at Scripps Mercy Hospital Chula Vista
- Are 18 years of age or older
- Have two or more chronic cardiovascular-metabolic conditions, such as obesity, diabetes, hypertension, dyslipidemia, ischemic heart disease, congestive heart failure, or other chronic coronary conditions.
- Have one or more behavioral health concerns such as feeling depressed, anxious, stressed, smoking, drinking too much alcohol, or having difficulty taking all your medications; and
- Have access to a telephone.

You cannot participate if you:

- Are pregnant
- Have a serious life-threatening condition
- Have a severe psychiatric or neurological/memory condition
- Are going to be discharged to inpatient rehabilitation or nursing care; or
- Do not speak Spanish or English.

**What happens in this research study?**
If you agree to participate in *Mi Puente* study, you will be asked to sign this consent form. A copy of the signed form will be given to you for your records. You will then be randomly assigned (like a flip of a coin) to be part of the study group *Mi Puente*, or the group that receives usual care. The study and follow-up period will last six months.

How will each group be treated?

*Usual Care Group:* If you are in the Usual Care group, your healthcare providers will follow all the usual discharge procedures. You will continue to receive medical care, have your medication reviewed, and receive care instructions while you are in the hospital. A team of healthcare professionals will help you with a discharge plan to follow once you leave the hospital.

*Mi Puente* Study Group: If you are in the *Mi Puente* study group, you will receive both usual care and support from a Behavioral Health Nurse and a Community Mentor.

The Behavioral Health Nurse will:

- Meet with you at least once while you are in the hospital.
- Find out about any language, social, or financial needs you may have.
- Help you manage your behavioral health concerns, such as:
  - Help you identify stress or other issues that may interfere with how you manage your health.
  - Discuss what to expect and what might motivate you to follow through with your discharge plan.
  - Create an action plan that takes your personal strengths and barriers into account.
  - Advise you on how to overcome problems and barriers while you seek help after leaving the hospital.

The Behavioral Health Nurse will review your "Personal Health Record" with you before you leave the hospital. She (or he) will telephone you within three days of leaving the hospital to make sure that you had a successful return home and to answer questions about your medications, symptoms, or referrals.

Before you leave the hospital, the Behavioral Health Nurse will try to introduce you to your "Community Mentor". Your Community Mentor will be your resource for up to 30 days after leaving the hospital. During your first meeting, the Behavioral Health Nurse will give you and your Community Mentor a copy of a form that outlines your action plan. The Community Mentor will schedule weekly follow-up telephone calls during the first and second week after you leave the hospital. If needed, they will call you again three and four weeks after you leave the hospital. If your Community Mentor believes you need medical advice, they will refer you to the Behavioral Health Nurse or to 911, if urgent.

**<u>What type of follow-up will I have</u>?**

All patients, both in the Usual Care group and in the Mi Puente group, will have the same surveys.

There will be three surveys, lasting about 20-30 minutes each, given by our bilingual research staff. The first survey will be done in person, before you leave the hospital, by a research staff member. After that, we will ask you to complete two more surveys by telephone at 3 and 6 months after you leave the hospital.

During these surveys, you will be asked questions about:

- Your background, such as where you were born, your employment status, and education
- Your quality of your life
- Any barriers to healthcare access
- Your knowledge, skill and confidence in managing your health and healthcare
- Resources you use to manage your chronic conditions

Medical Record Review

We will review your medical record for the next 6 months. We will check if you visit your doctor, go to the emergency department, or are admitted to the hospital again. If you need to see your doctor, we will review your health problems and your medications. We will also find out how long you stay in the hospital if you are admitted. We will review your medical records three times during the study:

- When you first agree to participate;
- At one month and;
- At six months after you leave the hospital.

**How will my information be used?**

The information we collect from you and the other people who take part in this study will be combined into one file. Members of the Mi Puente research team will then review this information to see:

- How often patients visited the Emergency Department or were admitted to the hospital;
- How often they sought other healthcare (routine or follow-up appointments);
- How they managed their health; and
- The quality of life patients had.

These results will be compared between the two groups, Usual Care and Mi Puente. We wish to find out if the program has any effect on how patients use healthcare and how they feel physically and emotionally. The results may be published in medical journals, but your name and personal information will never be shown in any report.

**What if you cannot reach me on the phone?**

If we cannot reach you, we will contact the relatives or others you name when you enroll in the study to help locate you. We will also attempt to search for your information through public directories.

**Is anything experimental in this study?**

All of the study techniques are well known and routinely used. None of the questionnaires or practices in this research is experimental. What is considered experimental is that we combine these techniques in a new way, to see whether this will be helpful to patients.

**Could I face any risks or discomforts?**

All of the techniques used in *Mi Puente* study are routine at doctors' offices and are considered safe. You may feel uncomfortable or embarrassed when you are asked sensitive questions or when you discuss your behavioral health needs or how you deal with your chronic condition. Well-trained research staff will make sure that you are as comfortable as possible. You are encouraged to complete all items and measures. You may refuse, however, to answer any question or participate in any surveys that makes you feel uncomfortable.

You may find it hard to give your time to our study. We know that your time is valuable and we will keep all the surveys as short as possible and conduct the follow-up surveys by phone at a time suitable to you. We will also give you reminder calls and send reminder letters to make communication easier and more successful.

**Is there a cost for participating in the study?**

There is no cost in money for participating in this study. The only cost is your time during contact with our research team.

Depending on your answers to the questions, our research staff may refer you to your healthcare provider for further examination, diagnosis, or treatment. Any cost related to diagnosis or treatment by your healthcare provider will be covered by either you or your health insurance. The *Mi Puente study* has no funds to pay for diagnostic procedures or treatment. This does not take away any of your legal rights.

**Will I be paid for participating?**

You will receive a $20 gift card when you complete each of the baseline and 3-month survey. You will receive a $25 gift card after you complete the 6-month survey. You can receive up to $65 in gift cards if you complete all of the surveys in the study.

**What if I refuse to participate in the study or wish to withdraw early?**

Taking part in this study is voluntary. You may decide not to join or you may leave the study at any time. Your decision will not result in any penalty or loss of benefits and it will have no effect on the quality of medical care you get. It will not affect your ability to get health care from Scripps Mercy Hospital Chula Vista. It will not affect any healthcare compensation or enrollment in any health plan. Taking part is completely up to you and if you choose to do so, you have the right to quit at any time.

If you decide to leave the study, we may still use the information collected about you unless you ask that we remove your records from the study files. If you choose to leave the study, you should call the *Mi Puente* Project Manager at the telephone number at the top of this form.

Taking part in this study may be stopped at any time by the investigators without your

consent. This may happen if it is considered best for your health or safety, if funding for the study ends, or for any other reason.

**What are my alternatives to joining the study?**
You may decide not to join this research study. You will then be offered the best-practice discharging procedures that are already in use. Your treatment will not be affected. Not joining is your alternative.

**What are my rights if I join?**

- You may call the Project Manager to ask any questions about this study. The telephone number is listed at the top of this form.
- You may decide not to take part in the study or you can decide to quit at any time after starting. Whatever you do, your medical care at Scripps Mercy Hospital Chula Vista or the community clinics will not be affected.
- For any questions about your rights, you may call the Scripps Office for the Protection of Research Subjects at (858) 678-6402. You should also read the *Experimental Subject's Bill of Rights*, which is on page 9 of this form.
- You retain all your legal rights whether you join this research study or not.
- You have the right to be told about any new information that might make you change your mind about participating in this study.

**What are my responsibilities if I join?**
If you join this study, you are expected to:

- Cooperate with the research staff.
- Keep or reschedule your study appointments.

**What if new information becomes available?**
If we have new information that may change your mind about taking part in the study, we will let you know as soon as possible. We will then ask you to tell us if you wish to continue or not.

**May I participate in other research studies, while taking part in *Mi Puente*?**
If you qualify for another research study, you are welcome to go on with whichever study you feel is better for you.

You may participate in any unrelated research study and may freely contact other study coordinators.

**What about confidentiality?**
Protecting your privacy is a top priority for *Mi Puente.* Any information we receive about you during this study will be treated as strictly confidential to the extent permitted by law. To make sure that the information you share is protected, a code number will be

assigned to you and your private information. This number will only be given to research staff and investigators of *Mi Puente*. Files linking names and other identifying information will be saved on a secure computer. We will use technology that prevents unauthorized individuals from accessing and reading this information. If your information is printed, it will be kept locked and accessible only to *Mi Puente* personnel.

When study results are published, your name and other identifying information will not be revealed. Results from this study and from your records may be reviewed and photocopied by federal regulatory agencies, such as the Office of Human Research Protection and the Institutional Review Boards of Scripps and/or San Diego State University. The researchers can share information without consent only in very special instances (for example, in case they believe that a person taking part in the study or some other individual is in serious danger of harm).

For more information, please read the ***Authorization to use your Private Health Information*** at the end of this form.

**Will Scripps Health, San Diego State University or the research investigators benefit from this study?**
Scripps Health, San Diego State University and the research investigators and staff will be paid to do this research under a research grant from the National Institutes of Health (NIH). Findings from this research study will also help to guide the care and treatment that is delivered to future patients.

**Questions and/or more information regarding this study:**
If you have any questions or would like more information at this moment about this research study, please ask. If you get to have any questions or concerns at any time while you are taking part in the study, please contact the Project Manager -- contact details are listed at the top of this form.

If you have questions regarding your rights as a participant in this study, you may contact the *Scripps Office for the Protection of Research Subjects* at (858) 678-6402.

**I agree to participate.**

*I have read and understood the explanation of the study. The study has also been explained to me by_________________________. I have had a chance to ask questions and have them answered to my satisfaction. I agree to take part in this study. I have not been forced or made to feel obligated to take part.*

*I have read the attached* ***Experimental Subject's Bill of Rights*** and the ***Authorization to use my Private Health Information*** *that contain some important information about research studies. I must sign this consent form, the* ***Experimental Subject's Bill of Rights*** *and the* ***Authorization to use my Private Health Information****. I will be given a signed copy of each to keep.*

| | | |
|---|---|---|
| ____________________________ | ____________________________ | ____________ |
| Printed Name of Subject | *Signature of Subject* | *Date* |

| | |
|---|---|
| ____________________________________ | ____________________________ |
| *Signature of person conducting the informed consent discussion* | Date |

_____________________________________________

Role of person named above in the research project

## EXPERIMENTAL SUBJECT'S BILL OF RIGHTS*

*If I am asked to consent to be a subject in a research study involving a medical experiment, or if I am asked to consent for someone else, I have the right to:*

*Learn the nature and purpose of the experiment (also called "study" or "clinical trial").*

*Receive an explanation of the procedures to be followed in the study, and any drug or device to be used.*

*Receive a description of any discomforts and risks that I could experience from the study.*

*Receive an explanation of any benefits I might expect from the study.*

*Learn about the risks and benefits of any other available procedures, drugs, or devices that might be helpful to me.*

*Learn what medical treatment will be made available to me if I should be injured because of the study.*

*Ask any questions about the study or the procedures involved.*

*Quit the study at any time, and my decision will not be used as an excuse to withhold necessary medical treatment.*

*Receive a copy of the signed and dated consent form.*

*Decide to consent or not to consent to a study without feeling forced or obligated.*

If I have questions about a research study, I can call the contact person listed on the consent form. If I have concerns about the research staff, or need more information about my rights as a subject, I can contact the Scripps Office for the Protection of Research Subjects, which protects volunteers in research studies. I may telephone the Office at **(858) 678-6402**, 8:00 a.m. to 4:00pm weekdays, or I may write to the Scripps Office for the Protection of Research Subjects, 4275 Campus Point Ct., CPB200, San Diego, CA 92121.

*By signing this document, I agree that I have read and received a copy of this Bill of Rights.*

___________________________________ ________________

Signature of Subject or Legal Representative Date

**California Health & Safety Code, Section 24172*

## Authorization to use your Private Health Information

**Name of Study:** *Mi Puente: My Bridge to Better Cardiometabolic Health and Well-Being*

**Principal Investigator:** *Athena Philis-Tsimikas, MD*

**What is private health information?**
Private health information is any information that can be traced back to you. We need your authorization (permission) to use your private health information in this research study. The private health information that we will use and share for this study includes:

- Your age, where you live, and how to contact you
- Information from your hospital and clinic records
- Answers to questions about your mental and physical health

**Who else will see my information?**

- Only the investigators named in the consent form and research staff that receives training in confidentiality procedures will see your information. In addition, Scripps committees that overview research to help protect people who join research studies may review your data if needed. Your name will not be used in any report that is written.
- If ***you*** share your information with people outside the research team, it will no longer be private.

**How long will Scripps use and share my information?**
Your information will be used and shared via reports and publications in aggregate (group) form (i.e., with no names or identifying information) for several years after the research is completed in 2020.

**What if I change my mind about sharing my research information?**
If you decide not to share your information anymore:

- The sponsor and the research team can continue to use any of the private information that they already have.
- You will no longer be a part of the research study.
- You will still get the same medical care that you have always had.

- 
- You must write to the investigator and tell her that you no longer want to share your information. Write to the investigator at:

Athena Philis-Tsimikas, MD
*10140 Campus Point Drive, Suite 200 San Diego, CA 92121*

**Do I have the right to see and copy my research information?**
You cannot see your research information while the study is going on, unless it is also being used for your health care. Once the study is over, you can ask to see any research information that is in your Medical Record that is kept at Scripps Whittier Diabetes Institute.

If you agree to share your information, you should sign this form below. You will be given a copy of this form.

******************************************************************

***I agree to share my information as described in this form***

_______________________________
Print your name

_______________________________ __________________
Sign your name Date

*If you have questions or concerns about your privacy and the use of your personal medical information, contact the investigator at the telephone number listed in the consent form.*
